# Association between prognostic factors and the outcomes of patients infected with SARS-CoV-2 harboring multiple spike protein mutations

**DOI:** 10.1038/s41598-021-00459-4

**Published:** 2021-11-01

**Authors:** Gunadi, Mohamad Saifudin Hakim, Hendra Wibawa, Marcellus, Ika Trisnawati, Endah Supriyati, Afiahayati, Riat El Khair, Kristy Iskandar, Siswanto, Irene, Nungki Anggorowati, Edwin Widyanto Daniwijaya, Dwi Aris Agung Nugrahaningsih, Yunika Puspadewi, Susan Simanjaya, Dyah Ayu Puspitarani, Hana Fauzyyah Hanifin, Alvina Alexandra Setiawan, Irene Tania, Cita Shafira Amalia, I. Putu Aditio Artayasa, Haries Rachman, Herdiyanto Mulyawan, Nur Rahmi Ananda, Eggi Arguni, Titik Nuryastuti, Tri Wibawa

**Affiliations:** 1 Pediatric Surgery Division, Department of Surgery/Genetics Working Group, Faculty of Medicine, Public Health and Nursing, Universitas Gadjah Mada, Jl. Kesehatan No. 1, Yogyakarta, 55281 Indonesia; 2 Department of Microbiology, Faculty of Medicine, Public Health and Nursing, Universitas Gadjah Mada, Yogyakarta, Indonesia; 3 Disease Investigation Center, Wates, Yogyakarta, Indonesia; 4 Genetics Working Group, Faculty of Medicine, Public Health and Nursing, Universitas Gadjah Mada, Yogyakarta, Indonesia; 5 Pulmonology Division, Department of Internal Medicine, Faculty of Medicine, Public Health and Nursing, Universitas Gadjah Mada/Dr. Sardjito Hospital, Yogyakarta, Indonesia; 6 Centre for Tropical Medicine, Faculty of Medicine, Public Health and Nursing, Universitas Gadjah Mada, Yogyakarta, Indonesia; 7 Department of Computer Science and Electronics Faculty of Mathematics and Natural Sciences, Universitas Gadjah Mada, Yogyakarta, Indonesia; 8 Department of Clinical Pathology and Laboratory Medicine, Faculty of Medicine, Public Health and Nursing, Universitas Gadjah Mada/Dr. Sardjito Hospital, Yogyakarta, 55281 Indonesia; 9 Department of Child Health/Genetics Working Group, Faculty of Medicine, Public Health and Nursing, Universitas Gadjah Mada/UGM Academic Hospital, Yogyakarta, Indonesia; 10 Department of Physiology, Faculty of Medicine, Public Health and Nursing, Universitas Gadjah Mada/UGM Academic Hospital, Yogyakarta, Indonesia; 11 Balai Besar Teknik Kesehatan Lingkungan Dan Pengendalian Penyakit, Yogyakarta, Yogyakarta, Indonesia; 12 Department of Anatomical Pathology/Genetics Working Group, Faculty of Medicine, Public Health and Nursing, Universitas Gadjah Mada, Yogyakarta, Indonesia; 13 Department of Microbiology, Faculty of Medicine, Public Health and Nursing, Universitas Gadjah Mada/UGM Academic Hospital, Yogyakarta, Indonesia; 14 Department of Pharmacology and Therapy/Genetics Working Group, Faculty of Medicine, Public Health and Nursing, Universitas Gadjah Mada, Yogyakarta, Indonesia; 15 Department of Child Health, Faculty of Medicine, Public Health and Nursing, Universitas Gadjah Mada/Dr. Sardjito Hospital, Yogyakarta, Indonesia

**Keywords:** Molecular medicine, Risk factors

## Abstract

The outcome of SARS-CoV-2 infection is determined by multiple factors, including the viral, host genetics, age, and comorbidities. This study investigated the association between prognostic factors and disease outcomes of patients infected by SARS-CoV-2 with multiple S protein mutations. Fifty-one COVID-19 patients were recruited in this study. Whole-genome sequencing of 170 full-genomes of SARS-CoV-2 was conducted with the Illumina MiSeq sequencer. Most patients (47%) had mild symptoms of COVID-19 followed by moderate (19.6%), no symptoms (13.7%), severe (4%), and critical (2%). Mortality was found in 13.7% of the COVID-19 patients. There was a significant difference between the age of hospitalized patients (53.4 ± 18 years) and the age of non-hospitalized patients (34.6 ± 19) (*p* = 0.001). The patients’ hospitalization was strongly associated with hypertension, diabetes, and anticoagulant and were strongly significant with the OR of 17 (95% CI 2–144; *p* = 0.001), 4.47 (95% CI 1.07–18.58; *p* = 0.039), and 27.97 (95% CI 1.54–507.13; *p* = 0.02), respectively; while the patients’ mortality was significantly correlated with patients’ age, anticoagulant, steroid, and diabetes, with OR of 8.44 (95% CI 1.5–47.49; *p* = 0.016), 46.8 (95% CI 4.63–472.77; *p* = 0.001), 15.75 (95% CI 2–123.86; *p* = 0.009), and 8.5 (95% CI 1.43–50.66; *p* = 0.019), respectively. This study found the clade: L (2%), GH (84.3%), GR (11.7%), and O (2%). Besides the D614G mutation, we found L5F (18.8%), V213A (18.8%), and S689R (8.3%). No significant association between multiple S protein mutations and the patients’ hospitalization or mortality. Multivariate analysis revealed that hypertension and anticoagulant were the significant factors influencing the hospitalization and mortality of patients with COVID-19 with an OR of 17.06 (95% CI 2.02–144.36; *p* = 0.009) and 46.8 (95% CI 4.63–472.77; *p* = 0.001), respectively. Moreover, the multiple S protein mutations almost reached a strong association with patients’ hospitalization (*p* = 0.07). We concluded that hypertension and anticoagulant therapy have a significant impact on COVID-19 outcomes. This study also suggests that multiple S protein mutations may impact the COVID-19 outcomes. This further emphasized the significance of monitoring SARS-CoV-2 variants through genomic surveillance, particularly those that may impact the COVID-19 outcomes.

## Introduction

After one year of the COVID-19 pandemic, SARS-CoV-2 has infected approximately 185 million people and causes 4 million deaths worldwide^1,2^. Indonesia has documented 2,379,397 COVID-19 cases and 62,908 deaths on July 7, 2021, and has become the highest cases country in the South-East Asian region^3^.

The outcome of SARS-CoV-2 infection is determined by multiple factors, including the viral and host genetics and age and comorbidities^4,5^. It is hypothesized that the host genetic factors might influence the outcome of SARS-CoV-2 infection. Three genes encoding the angiotensin-converting enzyme 2 (ACE2), the human leukocyte antigen (HLA), Toll-like receptor (TLR), and complement pathway are suggested to be the primary determinant of COVID-19 outcomes^6^. For viral genetic factors, a previous study indicated that variations within the ORF1ab (4715L) and S protein (614G) had a significant positive correlation with fatality rates of COVID-19^7^. The viral mutation may affect the presentation to MHC-I and MHC-II and consequently determine the magnitude of cellular immune responses^8^. The emergence of variants of concern (VOC) has attracted public health authorities to assess its impact on clinical presentation and severity. Indeed, the currently known VOCs (alpha, beta, gamma, and delta) have been associated with a possible increased risk of hospitalization and disease severity^9^.

SARS-CoV-2 has continuously and rapidly spread worldwide, providing a high opportunity for mutation events, especially on the S protein. However, the studies of the impact of multiple mutations within the spike (S) protein of SARS-CoV-2 on COVID-19 illness are limited. Thus, a comprehensive analysis of the impact of viral and various host factors on COVID-19 outcomes is highly needed. Our study determined the association between various prognostic factors and the disease outcomes of patients infected by SARS-CoV-2 harboring multiple S protein mutations.

## Material and methods

### Patients

This study was a retrospective study. We included all patients with COVID-19 from Yogyakarta and Central Java provinces, Indonesia, who sent their samples for whole genome sequencing into our institution from June to October 2020. The exclusion criteria were incomplete medical records.

Various clinical manifestations of COVID-19 have been noted, including asymptomatic until pneumonia with varying degrees. The degree of pneumonia of COVID-19 was classified according to the WHO classifications: (1) mild, without evidence of hypoxia or pneumonia; (2) moderate, pneumonia but not severe; (3) severe, pneumonia plus one of the following signs: respiratory rate > 30 breaths/minute (or based on age for children), severe respiratory distress, or SpO_2_ < 90% in room air; and (4) critical, Acute Respiratory Distress Syndrome (ARDS), sepsis, or septic shock, or other complications^10–12^.

### Prognostic factors

According to previous reports, we determined several prognostic factors to be associated with the outcomes of patients with COVID-19, including age, sex, comorbidity, smoking, and treatment^5,11^. We classified the outcomes of patients into two groups: hospitalized *vs*. non-hospitalized and survived *vs*. died.

The Medical and Health Research Ethics Committee of the Faculty of Medicine, Public Health and Nursing, Universitas Gadjah Mada/Dr. Sardjito Hospital approved this work (KE/FK/0563/EC/2020). All participants or guardians signed written informed consent for participating in this study.

### RNA extraction and whole-genome sequencing

RNA was extracted from all COVID-19 patients from Yogyakarta and Central Java provinces using QiAMP Viral RNA mini kit (Qiagen, Hilden, Germany), followed by real-time polymerase chain reaction (RT-PCR) using Real-Q 2019-nCoV Detection Kit (BioSewoom, Seoul, South Korea) with LightCycler® 480 Instrument II (Roche Diagnostics, Mannheim, Germany)^10,12^.

The double-stranded cDNA was synthesized using Maxima H Minus Double-Stranded cDNA Synthesis (Thermo Fisher Scientific, MA, United States), followed by purification of cDNA using a GeneJET PCR Purification Kit (Thermo Fisher Scientific, MA, United States) and library preparations using the Nextera DNA Flex for Enrichment using Respiratory Virus Oligos Panel. Next-generation sequencing (NGS) was performed to sequence the whole-genome of SARS-CoV-2 using the Illumina MiSeq instrument (Illumina, San Diego, CA, United States) with Illumina MiSeq reagents v3 150 cycles (2 × 75 cycles)^10,12^.

The assembly of our sample genomes was mapped into the reference genome from Wuhan, China (hCoV-19/Wuhan/Hu-1/2019, GenBank accession number: NC_045512.2) using Burrow-Wheeler Aligner (BWA) algorithm embedded in UGENE v. 1.30^13^. Single nucleotide polymorphisms (SNPs) were identified using the number of high confidence base calls (consensus sequence variations of the assembly) that disagree with the reference bases for the genome position of interest, followed by exporting all SNPs a vcf file and visualizing them in MS Excel^10,12^.

### Phylogenetic study

For the phylogenetic study, we utilized a dataset of 170 available SARS-CoV-2 genomes from our region and other countries that were retrieved from GISAID (Acknowledgment Table is provided in Supplementary Table 1), followed by multiple nucleotide sequence alignment using the MAFFT program (https://mafft.cbrc.jp/alignment/server/). Neighbour Joining statistical method with 1,000 bootstrap replications was used to construct a phylogenetic tree from 29.563 nucleotide length of the open reading frame (ORF) of the SARS-CoV-2 virus genome^14,15^. The Kimura 2-parameter method and the gamma distribution with estimated shape parameter (α) for the dataset were utilized to compute the evolutionary distances and model the rate variation among sites, respectively^16^. DAMBE version 7^17^ was used to calculate the estimation of the α gamma distribution, while MEGA version 10 (MEGA X)^18^ was utilized for all other analyses.

### Statistical analysis

The data were presented as frequency (percentage) and mean ± SD. The association between prognostic factors and outcomes was analyzed using Chi-square or Fisher exact tests with 95% confidence interval (CI), followed by a multivariate logistic regression test. The association was considered significant if the *p*-values of < 0.05. The IBM Statistical Package for the Social Sciences (SPSS) version 21 (Chicago, USA) was used to perform statistical analysis.

### Ethics approval and consent to participate

This study was approved by the Medical and Health Research Ethics Committee, Faculty of Medicine, Public Health and Nursing, Universitas Gadjah Mada/Dr. Sardjito Hospital, Yogyakarta, Indonesia (KE/FK/0563/EC/2020) and written informed consent was obtained. The research has been performed following the Declaration of Helsinki.

### Consent to publish

All participants or guardians signed written informed consent for participating in this study.

## Results

### Association between prognostic factors and hospitalization of patients with COVID-19

Among 51 patients, the clinical manifestations of COVID-19 were as follows: without any symptoms (13.7%), mild (47%), moderate (19.6%), severe (4%), critical (2%), and died (13.7%). The age of hospitalized patients (53.4 ± 18 years) was higher than non-hospitalized patients (34.6 ± 19) (*p* = 0.001) (Table 1).

**Table 1:** Association between prognostic factors and hospitalization of patients with COVID-19.

Characteristics	All (n = 51)	Hospitalized (n = 29) (n, %; mean ± SD)	Non-hospitalized (n = 22) (n, %; mean ± SD)	*p*-value	OR (95% CI)
RT-PCR Ct value		20.3 ± 4.2	18.9 ± **3.9**	0.26	
**Age (years)**		53.4 ± 18	34.6 ± 19	0.001*	
≥ 65	10	8 (27.6)	2 (9.1)	0.12	3.81 (0.72–20.16)
< 65	41	21 (72.4)	20 (90.9)		
**Sex**					
Male	30	19 (65.5)	11 (50)	0.27	1.9 (0.61–5.9)
Female	21	10 (34.5)	11 (50)		
**Comorbidity**					
Obesity	3	3 (10.3)	0	0.25	5.94 (0.29–121.31)
Diabetes	15	12 (41.4)	3 (13.6)	0.039*	4.47 (1.07–18.58)
Hypertension	14	13 (44.8)	1 (4.5)	0.001*	17 (2–144)
Cardiovascular disease	9	8 (27.6)	1 (4.5)	0.06	8 (0.92–69.72)
Chronic kidney disease	2	2 (6.9)	0	0.37	4.09 (0.19–89.65)
Smoking	4	1 (3.4)	3 (13.6)	0.21	0.23 (0.02–2.34)
**Therapy**					
ACEI/ARB	4	4 (13.8)	0	0.17	7.94 (0.33–66.14)
Anticoagulant	11	11 (37.9)	0	0.02*	27.97 (1.54–507.13)
Steroid	5	5 (17.2)	0	0.12	10.1 (0.53–193.23)

*Significant (*p* < 0.05).

*ACEI* angiotensin-converting enzyme inhibitors, *ARB* angiotensin receptor blocker, *CI* confidence interval, *OR* odds ratio.

### Association between prognostic factors and mortality of patients with COVID-19

A significant association between diabetes, hypertension, and anticoagulant therapy and the hospitalization of patients was found with *p*-value of 0.039 (OR = 4.47 [95% CI 1.07–18.58]), 0.001 (OR = 17 [95% CI 2–144]), and 0.02 (OR = 27.97 [95% CI 1.54–507.13]), respectively (Table 1). A strong association between patients’ age, diabetes, anticoagulant therapy, and steroid therapy and the mortality of patients was revealed with *p*-value of 0.016 (OR = 8.44 [95% CI 1.5–47.49]), 0.019 (OR = 8.5 [95% CI 1.43–50.66]), 0.001 (46.8 [95% CI 4.63–472.77]), and 0.009 (OR = 15.75 [95% CI 2–123.86]), respectively (Table 2).

**Table 2:** Association between prognostic factors and mortality of patients with COVID-19.

Characteristics	All (n = 51)	Died (n = 7) (n, %; mean ± SD)	Survived (n = 44) (n, %; mean ± SD)	*p*-value	OR (95% CI)
RT-PCR Ct value		18.7 ± 5.0	19.9 ± 3.9	0.57	
**Age (years)**		66.8 ± 14	41.8 ± 19	0.002*	
≥ 65	10	4 (57.1)	6 (13.6)	0.016*	8.44 (1.5–47.49)
< 65	41	3 (42.9)	38 (86.4)		
**Sex**					
Male	30	5 (71.4)	25 (56.8)	0.69	1.9 (0.33–10.88)
Female	21	2 (28.6)	19 (43.2)		
**Comorbidity**					
Obesity	3	1 (14.3)	2 (4.5)	0.34	3.5 (0.27–44.75)
Diabetes	15	5 (71.4)	10 (22.7)	0.019*	8.5 (1.43–50.66)
Hypertension	14	4 (57.1)	10 (22.7)	0.07	4.53 (0.87–23.72)
Cardiovascular disease	9	3 (42.9)	6 (13.6)	0.08	4.75 (0.84–26.71)
Chronic kidney disease	2	1 (14.3)	1 (2.3)	0.18	7.17 (0.39–130.31)
Smoking	4	0	4 (9.1)	0.74	0.6 (0.03–12.34)
**Therapy**					
ACEI/ARB	4	2 (28.6)	2 (4.5)	0.05	8.4 (0.96–73.43)
Anticoagulant	11	6 (85.7)	5 (11.4)	0.001*	46.8 (4.63–472.77)
Steroid	5	3 (42.9)	2 (4.5)	0.009*	15.75 (2–123.86)

*Significant (*p* < 0.05).

*ACEI* angiotensin-converting enzyme inhibitors, *ARB* angiotensin receptor blocker, *CI* confidence interval, *OR* odds ratio.

### Molecular and phylogenetic analysis

All viruses contained the D614G variant, except one isolate. Accordingly, the samples were classified as the following clade: L (2%), GH (84.3%), GR (11.7%), and O (2%). Besides the D614G mutation, the most common mutation in the S protein was L5F (18.8%), V213A (18.8%), and S689R (8.3%) (Table 3).

**Table 3:** Amino acid mutations observed in SARS-CoV-2 genomes collected from patients with COVID-19 in Yogyakarta and Central Java provinces.

	**5'UTR**					**NSP1-ORF1AB**						**NSP2-ORF1AB**					
VIRUS ID	**3**	**14**	**44**	**73**	**81**	**91**	**93**	**95**	**117**	**129**	**144**	**23**	**59**	**60**	**128**	**129**	**167**
NC_045512.2 WUHAN	G	L	*	P	R	E	E	I	A	K	D	D	A	W	Y	P	E
EPI_ISL_576130	G	L	*	P	**C**	E	E	I	A	K	D	D	A	W	Y	P	E
_EPI_ISL_902749	G	L	*	**S**	**C**	E	E	I	A	K	D	D	A	W	Y	P	E
_EPI_ISL_911709	G	L	*	P	**C**	E	E	I	A	K	D	D	A	W	Y	P	E
EPI_ISL_885142	G	L	*	P	**C**	E	E	I	A	K	D	D	A	W	Y	P	E
EPI_ISL_862040	G	L	*	P	**C**	E	E	I	A	K	D	D	A	W	Y	P	E
_EPI_ISL_877129	G	L	*	P	**C**	E	E	I	A	K	D	D	A	W	Y	P	E
_EPI_ISL_877128	G	L	*	P	**C**	E	E	I	A	K	D	D	A	W	Y	P	E
EPI_ISL_862041	G	L	*	P	**C**	E	E	I	A	K	D	D	A	W	Y	P	E
_EPI_ISL_891151	G	L	*	P	**C**	E	E	I	**V**	K	D	D	A	W	Y	P	E
_EPI_ISL_905731	G	L	*	P	**C**	E	E	I	A	K	D	D	A	W	Y	P	E
EPI_ISL_576383	G	L	*	P	**C**	E	E	I	A	K	D	D	A	W	Y	P	E
EPI_ISL_872190	G	L	*	P	**C**	E	E	I	A	K	D	D	A	W	Y	P	E
EPI_ISL_872189	G	**F**	*	P	**C**	E	E	I	A	K	D	D	A	W	Y	P	E
EPI_ISL_872188	G	L	*	P	**C**	E	E	I	A	K	D	D	A	W	Y	P	E
EPI_ISL_575331	G	L	*	P	**C**	E	E	I	A	K	D	D	A	W	Y	P	E
EPI_ISL_862039	G	L	*	P	**C**	E	E	I	A	K	D	D	A	W	Y	P	E
_EPI_ISL_902737	G	L	*	P	**C**	E	E	I	A	K	D	D	A	W	Y	P	E
_EPI_ISL_911707	G	L	*	P	**C**	E	E	I	A	K	D	D	A	W	Y	P	E
_EPI_ISL_877131	G	L	*	P	**C**	E	E	I	A	K	D	D	A	W	Y	P	E
_EPI_ISL_890185	G	L	*	P	**C**	E	E	I	A	K	D	D	A	W	**H**	P	E
_EPI_ISL_906050	G	L	*	P	**C**	E	E	I	A	K	D	D	A	W	Y	P	E
EPI_ISL_576116	G	L	*	P	**C**	E	E	I	A	K	D	D	A	W	Y	P	E
_EPI_ISL_877130	G	L	*	P	**C**	E	E	I	A	K	D	D	A	W	Y	P	E
_EPI_ISL_877126	G	L	*	P	**C**	E	E	I	A	K	D	D	A	W	Y	P	E
EPI_ISL_632936	G	L	*	P	**C**	E	E	I	A	K	D	D	A	W	Y	P	E
_EPI_ISL_985398	G	L	*	P	**C**	E	E	I	A	K	D	D	A	W	Y	P	E
_EPI_ISL_906052	G	L	*	P	**C**	E	E	I	A	K	D	D	A	W	Y	P	E
_EPI_ISL_890187	G	L	*	P	**C**	E	E	I	A	K	D	D	A	W	Y	P	E
_EPI_ISL_890186	G	L	*	P	**C**	E	E	I	A	K	D	D	A	W	Y	P	E
EPI_ISL_632937	G	L	*	P	**C**	E	E	I	A	K	D	D	A	W	Y	P	E
EPI_ISL_576115	G	L	*	P	**C**	E	E	I	A	K	D	D	A	W	Y	P	E
_EPI_ISL_877127	G	L	*	P	**C**	E	E	I	A	K	D	D	A	W	Y	P	E
_EPI_ISL_516800	G	L	*	P	**C**	E	E	I	A	K	D	D	A	W	Y	P	E
EPI_ISL_1005697	G	L	*	P	**C**	E	E	I	A	K	D	D	A	W	Y	P	E
EPI_ISL_576113	G	L	*	P	**C**	E	E	I	A	K	D	D	A	W	Y	P	E
EPI_ISL_1005696	G	L	*	P	**C**	E	E	I	A	K	D	D	A	W	Y	P	E
EPI_ISL_1005698	G	L	*	P	**C**	E	E	I	A	K	D	D	A	W	Y	P	E
EPI_ISL_985397	G	L	*	P	**C**	E	E	I	A	K	D	D	A	W	Y	**S**	E
EPI_ISL_985396	G	L	*	P	**C**	E	E	I	A	K	D	D	A	W	Y	P	**D**
EPI_ISL_525492	G	L	*	P	**C**	E	E	I	A	K	D	D	A	W	Y	P	E
EPI_ISL_576145	G	L	*	P	**C**	E	E	I	A	K	D	D	A	W	Y	P	E
EPI_ISL_906051	G	L	*	P	**C**	E	E	I	A	K	D	D	A	W	Y	P	E
EPI_ISL_610161	G	L	*	P	**C**	E	E	I	A	K	D	D	A	W	Y	P	E
EPI_ISL_516806	G	L	*	P	R	E	E	I	A	K	D	D	A	W	Y	P	E
EPI_ISL_576128	G	L	*	P	**C**	E	E	I	A	K	D	D	A	W	Y	P	E
EPI_ISL_576114	**C**	L	*	P	**C**	E	E	I	A	K	D	D	A	W	Y	P	E
EPI_ISL_610162	G	L	*	P	R	E	E	I	A	K	D	D	A	W	Y	P	E
EPI_ISL_516829	G	L	*	P	**C**	E	E	I	A	K	D	D	A	W	Y	P	E
EPI_ISL_1005695	G	L	*	P	**C**	E	E	I	A	K	D	D	A	W	Y	P	E
EPI_ISL_610155	G	L	**E**	P	**C**	**A**	**D**	**M**	A	*	D	**Y**	**D**	**G**	Y	**S**	E
EPI_ISL_610158	G	L	*	P	**C**	E	E	I	A	K	**N**	D	A	W	Y	P	E

	**NSP2-ORF1AB**									**NSP3-ORF1AB**							
VIRUS ID	**205**	**235**	**247**	**256**	**321**	**339**	**441**	**456**	**606**	**53**	**54**	**58**	**99**	**101**	**127**	**128**	**149**
NC_045512.2 WUHAN	A	G	A	T	Q	G	Y	K	N	F	A	A	A	H	T	Q	A
EPI_ISL_576130	A	G	A	**I**	**K**	G	Y	K	N	F	A	A	A	H	T	Q	A
_EPI_ISL_902749	A	G	A	T	Q	G	Y	K	N	F	A	**T**	A	H	T	Q	A
_EPI_ISL_911709	A	G	A	T	Q	G	Y	K	N	F	A	A	A	H	T	Q	A
EPI_ISL_885142	A	G	A	T	Q	G	Y	K	N	F	A	A	**S**	H	T	Q	A
EPI_ISL_862040	A	G	A	T	Q	G	Y	K	N	F	A	A	A	H	T	Q	A
_EPI_ISL_877129	A	G	A	T	Q	G	Y	K	N	F	A	A	A	H	T	Q	A
_EPI_ISL_877128	A	G	A	T	Q	G	Y	K	N	F	A	A	A	H	T	Q	A
EPI_ISL_862041	A	G	A	T	Q	G	Y	K	N	F	A	A	A	H	T	Q	A
_EPI_ISL_891151	A	G	A	T	Q	G	Y	K	N	F	A	A	A	**Y**	T	Q	A
_EPI_ISL_905731	A	G	A	T	Q	G	Y	K	N	F	A	**T**	A	H	T	Q	A
EPI_ISL_576383	**V**	G	A	T	Q	G	Y	K	N	F	A	A	A	H	T	Q	A
EPI_ISL_872190	A	G	A	T	Q	G	Y	K	N	**L**	A	A	A	H	T	Q	**V**
EPI_ISL_872189	A	G	A	T	Q	G	Y	K	N	F	A	A	A	H	T	Q	A
EPI_ISL_872188	A	G	A	T	Q	G	Y	K	N	F	A	A	A	H	T	Q	A
EPI_ISL_575331	A	G	A	T	Q	G	Y	K	N	F	A	A	A	H	T	Q	A
EPI_ISL_862039	A	G	A	T	Q	G	Y	K	N	F	A	A	**S**	H	T	Q	A
_EPI_ISL_902737	A	G	A	T	Q	G	Y	K	N	F	A	**T**	A	H	T	Q	A
_EPI_ISL_911707	A	G	A	T	Q	G	Y	K	N	F	A	A	A	H	T	Q	A
_EPI_ISL_877131	A	G	A	T	Q	G	Y	K	N	F	A	A	A	H	T	Q	A
_EPI_ISL_890185	A	G	A	T	Q	G	Y	K	N	F	A	A	A	H	T	Q	A
_EPI_ISL_906050	A	G	A	T	Q	G	Y	K	N	F	A	A	A	H	T	Q	A
EPI_ISL_576116	A	G	A	**I**	Q	G	Y	K	N	F	A	A	A	H	T	Q	A
_EPI_ISL_877130	A	G	A	T	Q	G	Y	K	N	F	A	A	A	H	T	Q	A
_EPI_ISL_877126	A	G	A	T	Q	G	Y	K	N	F	A	A	A	H	T	Q	A
EPI_ISL_632936	A	G	A	T	Q	G	Y	K	N	F	A	A	A	H	T	Q	A
_EPI_ISL_985398	A	**S**	A	T	Q	G	Y	K	N	F	A	A	A	H	T	Q	A
_EPI_ISL_906052	A	G	A	T	Q	G	Y	K	N	F	A	A	A	H	T	Q	A
_EPI_ISL_890187	A	G	A	T	Q	G	Y	K	N	F	**V**	A	A	H	T	Q	A
_EPI_ISL_890186	A	G	A	T	Q	G	Y	K	N	F	**V**	A	A	H	T	Q	A
EPI_ISL_632937	A	G	A	T	Q	G	Y	K	N	F	A	A	A	H	T	Q	A
EPI_ISL_576115	A	G	**V**	T	Q	G	Y	K	N	F	A	A	A	H	T	Q	A
_EPI_ISL_877127	A	G	A	T	Q	G	Y	K	N	F	A	A	A	H	T	Q	A
_EPI_ISL_516800	A	G	A	T	Q	G	Y	K	N	F	A	A	A	H	T	Q	A
EPI_ISL_1005697	A	G	A	T	Q	G	Y	K	N	F	**V**	A	A	H	T	Q	A
EPI_ISL_576113	A	G	**V**	T	Q	G	Y	K	N	F	A	A	A	H	T	Q	A
EPI_ISL_1005696	A	G	A	T	Q	G	Y	K	N	F	A	A	A	H	T	Q	A
EPI_ISL_1005698	A	G	A	T	Q	G	Y	**R**	N	F	A	A	A	H	T	Q	A
EPI_ISL_985397	A	G	A	T	Q	G	Y	K	N	F	A	A	A	H	T	Q	A
EPI_ISL_985396	A	G	A	T	Q	G	Y	K	N	F	A	A	A	H	T	Q	A
EPI_ISL_525492	A	G	A	T	Q	G	Y	K	N	F	A	A	A	H	T	Q	A
EPI_ISL_576145	A	G	A	T	Q	G	Y	K	N	F	A	A	A	H	T	Q	A
EPI_ISL_906051	A	G	A	T	Q	G	Y	K	N	F	A	A	A	H	T	Q	A
EPI_ISL_610161	A	G	A	T	Q	G	Y	K	N	F	A	A	A	H	T	Q	A
EPI_ISL_516806	A	G	A	T	Q	G	Y	K	N	F	A	A	A	H	T	Q	A
EPI_ISL_576128	A	G	A	**I**	**K**	G	Y	K	N	F	A	A	A	H	T	Q	A
EPI_ISL_576114	A	G	A	T	Q	G	Y	K	N	F	A	A	A	H	T	Q	A
EPI_ISL_610162	A	G	A	T	Q	G	Y	K	N	F	A	A	A	H	T	Q	A
EPI_ISL_516829	A	G	A	T	Q	G	Y	K	N	F	A	A	A	H	T	Q	A
EPI_ISL_1005695	A	G	A	T	Q	G	Y	K	N	F	A	A	A	H	T	Q	A
EPI_ISL_610155	A	G	A	T	Q	G	**N**	K	**I**	F	A	A	A	H	T	Q	A
EPI_ISL_610158	A	G	A	T	Q	**S**	Y	K	N	F	A	A	A	H	**N**	**K**	A

	**NSP3-ORF1AB**																
VIRUS ID	**196**	**228**	**231**	**299**	**325**	**469**	**663**	**679**	**822**	**945**	**1022**	**1115**	**1179**	**1197**	**1198**	**1230**	**1244**
NC_045512.2 WUHAN	M	V	A	V	V	V	G	P	P	K	T	D	A	S	T	S	L
EPI_ISL_576130	M	V	A	V	V	V	G	P	**L**	K	T	D	A	S	T	S	L
_EPI_ISL_902749	M	V	A	V	**F**	V	G	P	P	K	T	D	A	S	T	S	**F**
_EPI_ISL_911709	M	V	A	V	V	V	G	P	**L**	K	T	D	A	**T**	T	S	L
EPI_ISL_885142	M	V	**V**	V	V	V	G	P	P	K	T	D	A	S	T	S	L
EPI_ISL_862040	M	V	A	V	V	V	G	P	P	**N**	T	D	A	S	T	S	L
_EPI_ISL_877129	**L**	V	A	V	V	V	G	P	P	K	T	D	A	S	T	S	L
_EPI_ISL_877128	**L**	V	A	V	V	V	G	P	P	K	T	D	A	S	T	S	L
EPI_ISL_862041	M	V	A	V	V	V	G	P	P	K	T	D	A	S	T	S	L
_EPI_ISL_891151	M	V	A	V	V	V	G	P	P	K	T	D	A	S	T	S	L
_EPI_ISL_905731	M	V	A	V	**F**	V	G	P	P	K	T	D	A	S	T	S	**F**
EPI_ISL_576383	M	V	A	V	V	V	G	P	P	K	T	D	A	S	T	S	L
EPI_ISL_872190	M	V	A	V	V	V	G	P	**L**	K	T	D	A	S	T	S	L
EPI_ISL_872189	M	V	A	V	V	V	G	P	P	K	T	D	A	S	T	S	L
EPI_ISL_872188	M	V	A	V	V	**M**	G	P	P	K	T	D	A	S	T	S	L
EPI_ISL_575331	M	V	A	V	V	V	G	P	**L**	K	**I**	D	A	S	T	S	L
EPI_ISL_862039	M	V	**V**	V	V	V	G	P	P	K	T	D	A	S	T	S	L
_EPI_ISL_902737	M	V	A	V	**F**	V	G	P	P	K	T	D	A	S	T	S	**F**
_EPI_ISL_911707	M	V	A	V	V	V	G	P	**L**	K	T	D	A	**T**	T	S	L
_EPI_ISL_877131	**L**	V	A	V	V	V	G	P	P	K	T	D	A	S	T	S	L
_EPI_ISL_890185	M	V	A	V	V	V	G	P	P	K	T	D	A	S	T	S	L
_EPI_ISL_906050	M	V	A	V	V	V	G	P	P	K	T	D	A	S	T	S	L
EPI_ISL_576116	M	V	A	V	V	V	G	P	**L**	K	T	D	A	S	T	S	L
_EPI_ISL_877130	**L**	V	A	V	V	V	G	P	P	K	T	D	A	S	T	S	L
_EPI_ISL_877126	**L**	V	A	V	V	V	G	P	P	K	T	D	A	S	T	S	L
EPI_ISL_632936	M	V	A	V	V	V	G	P	**L**	K	T	D	**V**	S	T	S	L
_EPI_ISL_985398	M	V	A	V	V	V	G	P	**L**	K	T	D	A	S	T	S	L
_EPI_ISL_906052	M	V	A	V	V	V	G	P	P	K	T	D	A	S	T	S	L
_EPI_ISL_890187	M	V	A	V	V	V	G	P	**L**	K	T	D	A	S	T	S	L
_EPI_ISL_890186	M	V	A	V	V	V	G	P	**L**	K	T	D	A	S	T	S	L
EPI_ISL_632937	M	V	A	V	V	V	G	P	P	K	T	D	A	S	T	S	L
EPI_ISL_576115	M	V	A	V	V	V	G	P	**L**	K	T	D	A	S	T	S	L
_EPI_ISL_877127	**L**	V	A	V	V	V	G	P	P	K	T	D	A	S	T	S	L
_EPI_ISL_516800	M	V	A	V	V	V	G	P	**L**	K	T	D	A	S	T	S	L
EPI_ISL_1005697	M	V	A	V	V	V	G	P	**L**	K	T	D	A	S	T	S	L
EPI_ISL_576113	M	V	A	V	V	V	G	P	**L**	K	T	D	A	S	T	S	L
EPI_ISL_1005696	M	V	A	V	V	V	G	P	**L**	K	T	D	A	S	T	S	L
EPI_ISL_1005698	M	V	A	V	V	V	G	P	**L**	K	T	D	A	S	T	S	L
EPI_ISL_985397	M	V	A	V	V	V	G	P	**L**	K	T	D	A	S	T	S	L
EPI_ISL_985396	M	V	A	V	V	V	G	P	**L**	K	T	D	A	S	T	**F**	L
EPI_ISL_525492	M	V	A	V	V	V	G	P	**L**	K	T	D	A	S	T	S	L
EPI_ISL_576145	M	V	A	V	V	V	G	P	P	K	T	D	A	S	T	S	L
EPI_ISL_906051	M	V	A	V	V	V	G	P	P	K	T	D	A	S	T	S	L
EPI_ISL_610161	M	V	A	V	V	V	G	P	**L**	K	T	D	A	S	T	S	L
EPI_ISL_516806	M	V	A	V	V	V	G	P	P	K	T	D	A	S	T	S	L
EPI_ISL_576128	M	V	A	V	V	V	G	P	**L**	K	T	D	A	S	T	S	L
EPI_ISL_576114	M	V	A	**A**	V	V	G	P	**L**	K	T	D	A	S	T	S	L
EPI_ISL_610162	M	V	A	V	V	V	G	P	P	K	T	D	A	S	**K**	S	L
EPI_ISL_516829	M	V	A	V	V	V	G	**S**	P	K	T	D	A	S	T	S	L
EPI_ISL_1005695	M	V	A	V	V	V	G	P	P	K	T	D	A	S	T	S	L
EPI_ISL_610155	M	**L**	A	V	V	V	**S**	P	**L**	K	T	**Y**	A	S	T	S	L
EPI_ISL_610158	M	V	A	V	V	V	G	P	P	K	T	D	A	S	T	S	L

	**NSP3-ORF1AB**										**NSP4-ORF1AB**						**NSP5A-ORF1AB**
VIRUS ID	**1311**	**1354**	**1396**	**1419**	**1495**	**1596**	**1642**	**1665**	**1680**	**1770**	**117**	**143**	**231**	**361**	**383**	**386**	**12**
NC_045512.2 WUHAN	A	F	S	*	S	K	A	P	N	V	D	T	A	A	I	S	K
EPI_ISL_576130	A	F	S	*	S	K	A	P	N	V	D	T	A	A	I	S	K
_EPI_ISL_902749	A	F	S	*	S	K	A	P	N	V	D	T	A	A	I	S	K
_EPI_ISL_911709	A	F	S	*	S	K	A	P	N	V	D	T	A	A	I	S	K
EPI_ISL_885142	A	F	S	*	S	K	A	P	N	V	D	T	A	A	I	S	K
EPI_ISL_862040	A	F	**L**	*	S	K	A	P	N	V	D	T	A	A	I	S	K
_EPI_ISL_877129	A	F	S	*	S	K	A	P	N	V	D	T	A	A	**V**	S	K
_EPI_ISL_877128	A	F	S	*	S	K	A	P	N	V	D	T	A	A	**V**	S	K
EPI_ISL_862041	A	F	S	*	S	K	A	P	N	**F**	D	T	A	A	I	S	K
_EPI_ISL_891151	A	F	S	*	S	K	A	P	N	V	D	T	A	A	I	S	K
_EPI_ISL_905731	A	F	S	*	S	K	A	P	N	V	D	T	A	A	I	S	K
EPI_ISL_576383	A	F	S	*	S	K	A	P	N	V	D	T	A	A	I	S	K
EPI_ISL_872190	A	F	S	*	S	K	A	P	N	V	D	T	A	A	I	S	K
EPI_ISL_872189	**V**	F	S	*	S	K	A	P	N	V	D	**I**	A	A	I	S	K
EPI_ISL_872188	A	F	S	*	S	K	A	P	**S**	V	D	T	A	A	I	S	K
EPI_ISL_575331	A	F	S	*	S	K	A	P	N	V	D	T	A	A	I	S	K
EPI_ISL_862039	A	F	S	*	S	K	A	P	N	V	D	T	A	A	I	S	K
_EPI_ISL_902737	A	F	S	*	S	K	A	P	N	V	D	T	A	A	I	S	K
_EPI_ISL_911707	A	F	S	*	S	K	A	P	N	V	D	T	A	A	I	S	K
_EPI_ISL_877131	A	F	S	*	S	K	A	P	N	V	D	T	A	A	**V**	S	K
_EPI_ISL_890185	A	F	S	*	S	K	A	P	N	V	D	T	A	A	I	S	K
_EPI_ISL_906050	A	F	S	*	S	K	A	P	N	V	D	T	A	A	I	**N**	K
EPI_ISL_576116	A	F	S	*	S	K	A	P	N	V	D	T	A	A	I	S	**R**
_EPI_ISL_877130	A	F	S	*	S	K	A	P	N	V	D	T	A	A	**V**	S	K
_EPI_ISL_877126	A	F	S	*	S	K	A	P	N	V	D	T	A	A	**V**	S	K
EPI_ISL_632936	A	**C**	S	*	S	K	A	**L**	N	V	D	T	A	A	I	S	K
_EPI_ISL_985398	A	F	S	*	S	K	A	P	N	V	D	T	A	A	I	S	K
_EPI_ISL_906052	A	F	S	*	S	K	A	P	N	V	D	T	A	A	I	**N**	K
_EPI_ISL_890187	A	F	S	*	S	K	A	P	N	V	D	T	A	A	I	S	K
_EPI_ISL_890186	A	F	S	*	S	K	A	P	N	V	D	T	A	A	I	S	K
EPI_ISL_632937	A	F	S	*	S	K	A	P	N	V	D	T	A	A	I	S	K
EPI_ISL_576115	A	F	S	*	S	K	A	P	N	V	D	T	A	A	I	S	**R**
_EPI_ISL_877127	A	F	S	*	S	K	A	P	N	V	D	T	A	A	**V**	S	K
_EPI_ISL_516800	A	F	S	*	S	K	A	P	N	V	D	T	A	A	I	S	K
EPI_ISL_1005697	A	F	S	*	S	K	A	P	N	V	D	T	A	A	I	S	K
EPI_ISL_576113	A	F	S	*	S	K	A	P	N	V	D	T	A	A	I	S	**R**
EPI_ISL_1005696	A	F	S	*	S	K	A	P	N	V	D	T	A	A	I	S	K
EPI_ISL_1005698	A	F	S	*	S	K	A	P	N	V	D	T	A	A	I	S	K
EPI_ISL_985397	A	F	S	*	S	K	A	P	N	V	D	T	A	A	I	S	K
EPI_ISL_985396	A	F	S	*	**F**	K	**V**	P	N	V	**N**	T	A	**V**	I	S	K
EPI_ISL_525492	A	F	S	*	S	K	A	P	N	V	D	T	A	A	I	S	K
EPI_ISL_576145	A	F	S	*	S	K	A	P	N	V	D	T	A	A	I	S	K
EPI_ISL_906051	A	F	S	*	S	K	A	P	N	V	D	T	A	A	I	**N**	K
EPI_ISL_610161	A	F	S	*	S	K	A	P	N	V	D	T	A	A	I	S	**R**
EPI_ISL_516806	A	F	S	*	S	K	A	P	N	V	D	T	A	A	I	S	K
EPI_ISL_576128	A	F	S	*	S	K	A	P	N	V	D	T	A	A	I	S	K
EPI_ISL_576114	A	F	S	*	S	K	A	P	N	V	D	T	**V**	A	I	S	K
EPI_ISL_610162	A	F	S	*	S	K	A	P	N	V	D	T	A	A	I	S	K
EPI_ISL_516829	A	F	S	*	S	K	A	P	N	V	D	T	A	A	I	S	K
EPI_ISL_1005695	A	F	S	*	S	K	A	P	N	V	D	T	A	A	I	S	K
EPI_ISL_610155	A	F	S	*	S	**N**	A	P	N	V	D	T	A	A	I	S	K
EPI_ISL_610158	A	F	S	**L**	S	K	A	P	N	V	D	T	A	A	I	S	K

	**NSP5A-ORF1AB**					**NSP6-ORF1AB**		**NSP7-ORF1AB**		**NSP8-ORF1AB**				**NSP9-ORF1AB**			**NSP10-ORF1AB**
VIRUS ID	**49**	**87**	**163**	**184**	**284**	**8**	**83**	**37**	**50**	**16**	**21**	**100**	**117**	**36**	**42**	**83**	**73**
NC_045512.2 WUHAN	M	L	H	P	S	K	M	L	E	A	A	N	L	K	L	P	C
EPI_ISL_576130	M	L	H	P	S	K	M	L	E	A	A	N	L	K	L	P	C
_EPI_ISL_902749	M	L	H	P	S	K	M	L	E	A	A	N	L	K	L	P	C
_EPI_ISL_911709	M	L	H	P	S	K	**V**	L	E	A	A	N	L	K	L	P	C
EPI_ISL_885142	M	L	H	P	S	K	M	L	E	A	A	N	L	K	L	P	C
EPI_ISL_862040	M	L	H	P	**G**	**R**	M	L	**G**	A	A	N	L	K	L	P	C
_EPI_ISL_877129	M	L	H	P	S	K	M	L	E	A	A	N	L	K	L	P	C
_EPI_ISL_877128	M	L	H	P	S	K	M	L	E	A	A	N	L	K	L	P	C
EPI_ISL_862041	M	L	H	P	S	K	M	L	E	A	A	N	L	K	L	P	C
_EPI_ISL_891151	M	L	H	P	S	K	M	L	E	A	A	N	L	K	L	P	C
_EPI_ISL_905731	M	L	H	P	S	K	M	L	E	A	A	N	L	K	L	P	C
EPI_ISL_576383	M	L	H	P	S	K	M	L	E	A	A	N	L	K	L	P	C
EPI_ISL_872190	M	L	H	P	S	K	M	L	E	A	A	N	L	K	L	**L**	C
EPI_ISL_872189	M	L	H	P	S	K	M	L	E	**V**	A	N	L	K	L	P	C
EPI_ISL_872188	M	L	H	P	S	K	M	L	E	A	A	N	L	K	L	P	C
EPI_ISL_575331	M	L	H	P	S	K	M	L	E	A	A	N	L	K	L	P	C
EPI_ISL_862039	M	L	H	P	S	K	M	L	E	A	A	N	L	K	L	P	C
_EPI_ISL_902737	M	L	H	P	S	K	M	L	E	A	A	N	L	K	L	P	C
_EPI_ISL_911707	M	L	H	P	S	K	**V**	L	E	A	A	N	L	K	L	P	C
_EPI_ISL_877131	M	L	H	P	S	K	M	L	E	A	A	N	L	K	L	P	C
_EPI_ISL_890185	M	L	H	P	S	K	M	L	E	A	A	N	L	K	L	P	C
_EPI_ISL_906050	M	**F**	H	P	S	K	M	L	E	A	A	N	L	K	L	P	C
EPI_ISL_576116	M	L	H	P	S	K	M	L	E	A	A	N	L	K	L	P	C
_EPI_ISL_877130	M	L	H	P	S	K	M	L	E	A	A	N	L	K	L	P	C
_EPI_ISL_877126	M	L	H	P	S	K	M	L	E	A	A	N	L	K	L	P	C
EPI_ISL_632936	M	L	H	P	S	K	M	L	E	A	A	N	L	K	L	P	C
_EPI_ISL_985398	M	L	H	P	S	K	M	L	E	A	A	**S**	L	K	L	P	C
_EPI_ISL_906052	M	**F**	H	P	S	K	M	L	E	A	A	N	L	K	L	P	C
_EPI_ISL_890187	M	L	H	P	S	K	M	L	E	A	A	N	L	K	L	P	C
_EPI_ISL_890186	M	L	H	P	S	K	M	L	E	A	A	N	L	K	L	P	C
EPI_ISL_632937	M	L	H	P	S	K	M	L	E	A	A	N	L	K	L	P	C
EPI_ISL_576115	M	L	H	P	S	K	M	L	E	A	A	N	L	K	L	P	C
_EPI_ISL_877127	M	L	H	P	S	K	M	L	E	A	A	N	L	K	L	P	C
_EPI_ISL_516800	M	L	H	P	S	K	M	L	E	A	A	N	L	K	L	P	C
EPI_ISL_1005697	M	L	H	P	S	K	M	L	E	A	A	N	L	K	L	P	C
EPI_ISL_576113	M	L	H	P	S	K	M	L	E	A	A	N	L	K	L	P	C
EPI_ISL_1005696	M	L	H	P	S	K	M	L	E	A	A	N	L	K	L	P	C
EPI_ISL_1005698	M	L	H	P	S	K	M	L	E	A	A	N	L	K	L	P	C
EPI_ISL_985397	M	L	H	P	S	K	M	L	E	A	A	N	L	K	L	P	C
EPI_ISL_985396	M	L	H	P	S	K	M	**F**	E	A	A	N	L	K	L	P	C
EPI_ISL_525492	M	L	H	P	S	K	M	L	E	A	A	N	L	K	L	P	C
EPI_ISL_576145	M	L	H	**S**	S	K	M	L	E	A	A	N	L	K	L	P	C
EPI_ISL_906051	M	**F**	H	P	S	K	M	L	E	A	A	N	L	K	L	P	C
EPI_ISL_610161	M	L	H	P	S	K	M	L	E	A	A	N	L	K	L	P	C
EPI_ISL_516806	**I**	L	H	P	S	K	M	L	E	A	A	N	L	K	L	P	C
EPI_ISL_576128	M	L	H	P	S	K	M	L	E	A	A	N	L	K	L	P	C
EPI_ISL_576114	M	L	H	P	S	K	M	L	E	A	A	N	L	K	L	P	C
EPI_ISL_610162	M	L	H	P	S	K	M	**F**	E	A	**T**	N	L	K	**F**	P	C
EPI_ISL_516829	M	L	H	P	S	K	M	L	E	A	A	N	L	K	L	P	C
EPI_ISL_1005695	M	L	H	P	S	K	M	L	E	A	A	N	L	K	L	P	C
EPI_ISL_610155	M	L	H	P	S	K	M	L	E	A	A	N	L	K	L	P	C
EPI_ISL_610158	M	L	**N**	P	S	K	M	L	E	A	A	N	**M**	**R**	L	P	*

	**NSP12-ORF1AB**																
VIRUS ID	**88**	**143**	**218**	**239**	**255**	**265**	**314**	**370**	**425**	**465**	**469**	**520**	**587**	**598**	**647**	**794**	**850**
NC_045512.2 WUHAN	A	C	P	T	P	D	P	A	S	E	K	A	G	S	A	T	F
EPI_ISL_576130	A	C	P	T	P	D	**L**	A	S	E	K	A	G	S	A	T	F
_EPI_ISL_902749	A	C	P	T	P	D	**L**	A	S	E	K	A	G	S	A	T	F
_EPI_ISL_911709	A	C	P	T	P	D	**L**	A	S	E	K	A	G	S	A	T	F
EPI_ISL_885142	A	C	P	T	P	D	**L**	A	S	E	K	**V**	G	S	A	T	F
EPI_ISL_862040	A	C	P	T	P	D	**L**	A	S	E	K	A	G	S	A	T	F
_EPI_ISL_877129	A	C	**L**	T	P	D	**L**	A	S	E	K	A	G	S	A	**I**	F
_EPI_ISL_877128	A	C	**L**	T	P	D	**L**	A	S	E	K	A	G	S	A	**I**	F
EPI_ISL_862041	A	C	P	T	P	D	**L**	**T**	S	E	K	A	G	**N**	A	T	F
_EPI_ISL_891151	A	C	P	T	P	D	**L**	A	S	E	**N**	A	G	S	A	T	F
_EPI_ISL_905731	A	C	P	T	P	D	**L**	A	S	E	K	A	G	S	A	T	F
EPI_ISL_576383	A	C	P	T	P	D	**L**	A	S	E	K	A	G	S	A	T	F
EPI_ISL_872190	A	C	P	T	P	D	**L**	A	S	E	K	A	G	S	A	T	F
EPI_ISL_872189	A	C	P	T	P	D	**L**	A	S	E	K	A	G	S	A	T	F
EPI_ISL_872188	A	C	P	T	P	D	**L**	A	S	E	K	A	G	S	A	T	F
EPI_ISL_575331	A	C	P	T	P	D	**L**	A	S	E	K	A	G	S	A	T	F
EPI_ISL_862039	A	C	P	T	P	D	**L**	A	S	E	K	**V**	G	S	A	T	F
_EPI_ISL_902737	A	C	P	T	P	D	**L**	A	S	E	K	A	G	S	A	T	F
_EPI_ISL_911707	A	C	P	T	P	D	**L**	A	S	E	K	A	G	S	A	T	F
_EPI_ISL_877131	A	C	**L**	T	P	D	**L**	A	S	E	K	A	G	S	A	**I**	F
_EPI_ISL_890185	A	C	P	T	P	D	**L**	A	S	E	K	A	G	S	A	T	F
_EPI_ISL_906050	A	C	**L**	T	P	D	**L**	A	S	E	K	A	G	S	A	**I**	F
EPI_ISL_576116	A	C	P	**I**	P	D	**L**	A	S	E	K	A	G	S	A	T	F
_EPI_ISL_877130	A	C	**L**	T	P	D	**L**	A	S	E	K	A	G	S	A	**I**	F
_EPI_ISL_877126	A	C	**L**	T	P	D	**L**	A	S	E	K	A	G	S	A	**I**	F
EPI_ISL_632936	A	C	P	T	P	D	**L**	A	S	E	K	A	G	S	A	T	F
_EPI_ISL_985398	A	C	P	T	P	D	**L**	A	S	E	K	A	G	S	A	T	F
_EPI_ISL_906052	A	C	**L**	T	P	D	**L**	A	S	E	K	A	G	S	A	**I**	F
_EPI_ISL_890187	A	C	P	T	P	D	**L**	A	S	E	K	A	G	S	A	T	F
_EPI_ISL_890186	A	C	P	T	P	D	**L**	A	S	E	K	A	G	S	A	T	F
EPI_ISL_632937	A	C	P	T	P	D	P	A	S	E	K	A	G	S	A	T	F
EPI_ISL_576115	A	C	P	**I**	P	D	**L**	A	S	E	K	A	G	S	A	T	F
_EPI_ISL_877127	A	C	**L**	T	P	D	**L**	A	S	E	K	A	G	S	A	**I**	F
_EPI_ISL_516800	A	C	P	T	P	D	**L**	A	S	E	K	A	G	S	A	T	F
EPI_ISL_1005697	A	C	P	T	P	D	**L**	A	S	E	K	A	G	S	A	T	F
EPI_ISL_576113	A	C	P	**I**	P	D	**L**	A	S	E	K	A	G	S	A	T	F
EPI_ISL_1005696	A	C	P	T	P	D	**L**	A	**F**	E	K	A	G	S	A	T	F
EPI_ISL_1005698	A	C	P	T	P	D	**L**	A	**F**	E	K	A	G	S	A	T	F
EPI_ISL_985397	A	C	P	T	P	D	**L**	A	S	E	K	A	G	S	A	T	F
EPI_ISL_985396	A	C	P	T	P	D	**L**	A	S	E	K	A	**S**	S	A	T	F
EPI_ISL_525492	A	C	P	T	P	D	**L**	A	S	E	K	A	G	S	A	T	F
EPI_ISL_576145	A	C	P	T	P	D	**L**	A	S	E	K	A	G	S	A	T	F
EPI_ISL_906051	A	C	**L**	T	P	D	**L**	A	S	E	K	A	G	S	A	**I**	F
EPI_ISL_610161	A	C	P	**I**	P	D	**L**	A	S	E	K	A	G	S	A	T	F
EPI_ISL_516806	A	C	P	T	P	D	P	A	S	E	K	A	G	S	A	T	F
EPI_ISL_576128	A	C	P	T	P	D	**L**	A	S	E	K	A	G	S	A	T	F
EPI_ISL_576114	A	C	P	T	P	D	**L**	A	S	E	K	A	G	S	A	T	F
EPI_ISL_610162	**V**	C	P	T	P	D	P	A	S	E	K	A	G	S	A	T	F
EPI_ISL_516829	A	C	P	T	P	D	**L**	A	S	E	K	A	G	S	**S**	T	F
EPI_ISL_1005695	A	C	P	T	P	D	**L**	A	S	**V**	K	A	G	S	A	T	F
EPI_ISL_610155	A	**S**	P	T	**L**	**Y**	**L**	A	**T**	E	K	A	G	S	A	T	F
EPI_ISL_610158	A	C	P	T	P	D	P	A	S	E	K	A	G	S	A	T	**C**

	**NSP12-ORF1AB**		**NSP13-ORF1AB**										**NSP14A-ORF1AB**				
VIRUS ID	**883**	**897**	**9**	**127**	**153**	**169**	**236**	**469**	**507**	**568**	**576**	**588**	**10**	**125**	**153**	**203**	**225**
NC_045512.2 WUHAN	H	M	N	T	T	V	S	A	R	A	M	T	D	I	M	P	A
EPI_ISL_576130	**Y**	M	N	T	T	V	S	A	R	A	M	T	D	I	M	P	A
_EPI_ISL_902749	H	M	N	T	T	V	S	A	R	A	M	**I**	D	I	M	P	A
_EPI_ISL_911709	H	M	N	T	T	V	S	A	R	A	M	T	D	I	M	P	A
EPI_ISL_885142	H	M	N	T	T	V	S	A	R	A	M	T	D	I	M	P	A
EPI_ISL_862040	H	M	N	T	T	V	S	A	R	A	M	T	D	I	M	P	A
_EPI_ISL_877129	H	M	N	T	T	V	S	A	R	A	M	T	D	I	M	P	A
_EPI_ISL_877128	H	M	N	T	T	V	S	A	R	A	M	T	D	I	M	P	A
EPI_ISL_862041	H	M	N	T	T	V	S	A	R	A	M	T	D	I	M	P	A
_EPI_ISL_891151	H	M	N	T	T	V	S	A	R	A	M	T	D	I	M	P	A
_EPI_ISL_905731	H	M	N	T	T	V	S	A	R	A	M	**I**	D	I	M	P	A
EPI_ISL_576383	H	M	N	T	T	V	S	A	R	A	M	T	D	I	M	P	A
EPI_ISL_872190	H	M	N	T	T	V	S	A	R	A	M	T	D	I	M	P	A
EPI_ISL_872189	H	M	N	T	T	V	S	A	R	A	M	T	D	I	M	P	A
EPI_ISL_872188	H	M	N	T	T	V	S	**S**	R	A	M	T	D	I	M	P	A
EPI_ISL_575331	H	M	N	T	T	V	S	A	R	A	M	T	D	I	M	P	A
EPI_ISL_862039	H	M	N	T	T	V	S	A	R	A	M	T	D	I	M	P	A
_EPI_ISL_902737	H	M	N	T	T	V	S	A	R	A	M	**I**	D	I	M	P	A
_EPI_ISL_911707	H	M	N	T	T	V	S	A	R	A	M	T	D	I	M	P	A
_EPI_ISL_877131	H	M	N	T	T	V	S	A	R	A	M	T	D	I	M	P	A
_EPI_ISL_890185	H	M	N	T	T	V	S	A	R	A	M	T	D	I	M	P	**T**
_EPI_ISL_906050	H	M	N	T	T	V	S	A	R	A	M	T	D	I	M	P	A
EPI_ISL_576116	H	M	N	T	T	V	S	A	R	A	M	T	D	I	M	P	A
_EPI_ISL_877130	H	M	N	T	T	V	S	A	R	A	M	T	D	I	M	P	A
_EPI_ISL_877126	H	M	N	T	T	V	S	A	R	A	M	T	D	I	M	P	A
EPI_ISL_632936	H	M	N	T	T	V	S	A	R	A	M	T	D	I	M	P	A
_EPI_ISL_985398	H	M	N	T	T	V	S	A	R	**V**	M	T	D	**V**	M	P	A
_EPI_ISL_906052	H	M	N	T	T	V	S	A	R	A	M	T	D	I	M	P	A
_EPI_ISL_890187	H	M	N	T	T	V	S	A	R	A	M	T	D	I	M	P	A
_EPI_ISL_890186	H	M	N	T	T	V	S	A	R	A	M	T	D	I	M	P	A
EPI_ISL_632937	H	M	N	T	T	V	S	A	R	A	M	T	D	I	M	P	A
EPI_ISL_576115	H	M	N	T	T	V	S	A	R	A	M	T	D	I	M	P	A
_EPI_ISL_877127	H	M	N	T	T	V	S	A	R	A	M	T	D	I	M	P	A
_EPI_ISL_516800	H	M	N	T	T	V	S	A	R	A	M	T	D	I	M	P	A
EPI_ISL_1005697	H	M	N	T	T	V	S	A	R	A	M	T	D	I	M	P	A
EPI_ISL_576113	H	M	N	T	T	V	S	A	R	A	M	T	D	I	M	P	A
EPI_ISL_1005696	H	M	N	T	T	V	S	A	R	A	M	T	D	I	M	P	A
EPI_ISL_1005698	H	M	N	T	T	V	S	A	R	A	M	T	D	I	M	P	A
EPI_ISL_985397	H	M	N	T	T	V	S	A	R	A	M	T	D	I	M	P	A
EPI_ISL_985396	H	M	N	T	T	V	S	A	R	A	M	T	D	I	M	P	A
EPI_ISL_525492	H	M	N	T	T	V	S	A	R	A	M	T	D	I	M	P	A
EPI_ISL_576145	H	M	N	T	T	V	S	A	R	A	M	T	D	I	M	P	A
EPI_ISL_906051	H	M	N	T	T	V	S	A	R	A	M	T	D	I	M	P	A
EPI_ISL_610161	H	M	N	T	T	**F**	S	A	R	A	M	T	D	I	M	P	A
EPI_ISL_516806	H	M	N	T	T	V	S	A	R	A	M	T	D	I	M	P	A
EPI_ISL_576128	**Y**	M	N	T	T	V	S	A	R	A	M	T	D	I	M	P	A
EPI_ISL_576114	H	M	N	**I**	T	V	S	A	R	A	M	T	D	I	M	**L**	A
EPI_ISL_610162	H	**V**	N	T	**I**	V	S	A	R	A	M	T	D	I	M	P	A
EPI_ISL_516829	H	M	N	T	T	V	S	A	R	A	**I**	T	D	I	M	P	A
EPI_ISL_1005695	H	M	N	T	T	V	S	A	R	A	M	T	D	I	M	P	A
EPI_ISL_610155	H	M	N	T	T	V	**C**	A	R	A	M	T	D	I	**K**	P	A
EPI_ISL_610158	H	M	**K**	T	T	V	S	A	**K**	A	M	T	**E**	I	M	P	A

	**NSP14A-ORF1AB**		**NSP15-ORF1AB**									**NSP16-ORF1AB**					
VIRUS ID	**327**	**479**	**58**	**74**	**171**	**236**	**275**	**276**	**279**	**331**	**337**	**17**	**35**	**125**	**126**	**127**	**165**
NC_045512.2 WUHAN	P	L	W	*	A	V	V	*	F	L	H	M	T	G	L	S	E
EPI_ISL_576130	P	L	W	*	A	V	V	*	F	L	H	M	T	G	L	S	E
_EPI_ISL_902749	P	L	W	*	A	V	V	*	F	L	H	M	T	G	L	S	E
_EPI_ISL_911709	P	L	W	*	A	V	V	*	F	L	H	M	T	G	L	S	E
EPI_ISL_885142	P	L	W	*	A	**F**	V	*	F	L	H	**I**	**I**	G	L	S	E
EPI_ISL_862040	P	L	**F**	*	A	V	V	*	F	L	H	M	T	G	L	S	E
_EPI_ISL_877129	P	L	W	*	A	V	V	*	F	L	H	M	T	G	L	S	E
_EPI_ISL_877128	P	L	W	*	A	V	V	*	F	L	H	M	T	G	L	S	E
EPI_ISL_862041	P	L	W	*	A	V	V	*	F	L	H	M	T	G	L	S	E
_EPI_ISL_891151	P	L	W	*	A	V	V	*	F	L	H	M	T	G	L	S	E
_EPI_ISL_905731	P	L	W	*	A	V	V	*	F	L	H	M	T	G	L	S	E
EPI_ISL_576383	P	L	W	*	A	V	V	*	F	L	H	M	T	G	L	S	E
EPI_ISL_872190	P	L	W	*	A	V	V	*	F	L	H	M	T	G	L	S	E
EPI_ISL_872189	P	L	W	*	A	V	V	*	F	L	H	M	T	G	L	S	E
EPI_ISL_872188	**L**	L	W	*	A	V	V	*	F	L	H	M	T	G	**F**	S	E
EPI_ISL_575331	P	L	W	*	A	V	V	*	F	L	H	M	T	G	L	S	E
EPI_ISL_862039	P	L	W	*	A	**F**	V	*	F	L	H	**I**	**I**	G	L	S	E
_EPI_ISL_902737	P	L	W	*	A	V	V	*	F	L	H	M	T	G	L	S	E
_EPI_ISL_911707	P	L	W	*	A	V	V	*	F	L	H	M	T	G	L	S	E
_EPI_ISL_877131	P	L	W	*	A	V	V	*	F	L	H	M	T	G	L	S	E
_EPI_ISL_890185	P	L	W	*	A	V	V	*	F	L	H	M	T	G	L	S	E
_EPI_ISL_906050	P	L	W	*	A	V	V	*	F	L	H	M	T	G	L	S	E
EPI_ISL_576116	P	L	W	*	A	V	V	*	F	L	H	M	T	G	L	S	E
_EPI_ISL_877130	P	L	W	*	A	V	V	*	F	L	H	M	T	G	L	S	E
_EPI_ISL_877126	P	L	W	*	A	V	V	*	F	L	H	M	T	G	L	S	E
EPI_ISL_632936	P	L	W	*	A	V	V	*	F	L	H	M	T	G	L	S	E
_EPI_ISL_985398	P	L	W	*	A	V	V	*	F	L	H	M	T	G	L	S	E
_EPI_ISL_906052	P	L	W	*	A	V	V	*	F	L	H	M	T	G	L	S	E
_EPI_ISL_890187	P	L	W	*	A	V	V	*	F	L	H	M	T	G	L	S	E
_EPI_ISL_890186	P	L	W	*	A	V	V	*	F	L	H	M	T	G	L	S	E
EPI_ISL_632937	P	L	W	*	A	V	V	*	F	L	H	M	T	G	L	S	E
EPI_ISL_576115	P	L	W	*	A	V	V	*	F	L	H	M	T	G	L	S	E
_EPI_ISL_877127	P	L	W	*	A	V	V	*	F	L	H	M	T	G	L	S	E
_EPI_ISL_516800	P	L	W	*	A	V	V	*	F	L	H	M	T	G	L	S	E
EPI_ISL_1005697	P	L	W	*	A	V	V	*	F	L	H	M	T	G	L	S	E
EPI_ISL_576113	P	L	W	*	A	V	V	*	F	L	H	M	T	G	L	S	E
EPI_ISL_1005696	P	L	W	*	A	V	**F**	*	F	**F**	H	M	T	G	L	S	E
EPI_ISL_1005698	P	L	W	*	**V**	V	V	*	F	L	H	M	T	G	L	S	E
EPI_ISL_985397	P	L	W	*	A	V	V	*	F	L	H	M	T	G	L	S	E
EPI_ISL_985396	P	L	W	*	A	V	V	*	F	L	H	M	T	G	L	S	E
EPI_ISL_525492	P	L	W	*	A	V	V	*	F	L	H	M	T	G	L	S	E
EPI_ISL_576145	P	L	W	*	A	V	V	*	F	L	**Y**	M	T	G	L	S	E
EPI_ISL_906051	P	L	W	*	A	V	V	*	F	L	H	M	T	G	L	S	E
EPI_ISL_610161	P	L	W	*	A	V	V	*	F	L	H	M	T	G	L	S	E
EPI_ISL_516806	P	L	W	*	A	V	V	*	F	L	H	M	T	G	L	S	E
EPI_ISL_576128	P	L	W	*	A	V	V	*	F	L	H	M	T	G	L	S	E
EPI_ISL_576114	P	L	W	*	A	V	V	*	F	L	H	M	T	G	L	S	E
EPI_ISL_610162	P	L	W	*	A	V	V	*	F	L	H	M	T	G	L	S	E
EPI_ISL_516829	P	L	W	*	A	V	V	*	F	L	H	M	T	G	L	S	E
EPI_ISL_1005695	P	L	W	*	A	V	V	*	F	L	H	M	T	G	L	S	E
EPI_ISL_610155	P	**V**	W	*	A	V	V	*	F	L	H	M	T	G	L	S	E
EPI_ISL_610158	P	L	W	**K**	A	V	V	**L**	**L**	L	H	M	T	**A**	L	*	**Q**

	NSP16-ORF1AB	**SPIKE GLYCOPROTEIN**															
VIRUS ID	**222**	**5**	**12**	**14**	**16**	**18**	**29**	**61**	**83**	**127**	**145**	**157**	**186**	**193**	**213**	**214**	**218**
NC_045512.2 WUHAN	Y	L	S	Q	V	L	T	N	V	V	Y	F	F	V	V	R	Q
EPI_ISL_576130	Y	**F**	S	Q	V	L	T	N	V	V	Y	F	F	V	V	R	Q
_EPI_ISL_902749	Y	L	S	Q	V	L	T	N	V	V	Y	F	F	V	V	R	Q
_EPI_ISL_911709	Y	L	S	Q	V	L	T	N	V	V	Y	F	F	V	V	R	Q
EPI_ISL_885142	Y	L	S	Q	V	L	T	N	V	V	Y	F	F	V	V	R	Q
EPI_ISL_862040	Y	L	S	Q	V	L	T	N	V	V	Y	F	F	V	V	R	Q
_EPI_ISL_877129	Y	**F**	S	Q	V	L	T	N	V	V	Y	F	F	V	V	R	Q
_EPI_ISL_877128	Y	**F**	S	Q	V	L	T	N	V	V	Y	F	F	V	V	R	Q
EPI_ISL_862041	Y	L	S	Q	V	L	T	N	V	V	Y	F	F	V	**L**	R	Q
_EPI_ISL_891151	Y	L	S	Q	V	L	T	N	V	V	Y	F	F	V	V	R	Q
_EPI_ISL_905731	Y	L	S	Q	V	L	T	N	V	V	Y	F	F	V	V	R	Q
EPI_ISL_576383	**C**	L	S	Q	V	L	T	N	V	V	Y	F	F	V	V	R	Q
EPI_ISL_872190	Y	L	S	Q	V	L	T	N	V	V	Y	F	F	V	V	R	Q
EPI_ISL_872189	Y	L	S	Q	V	**F**	T	N	V	V	Y	F	F	V	V	R	Q
EPI_ISL_872188	Y	L	S	Q	V	L	T	N	V	V	Y	F	F	V	V	R	**R**
EPI_ISL_575331	Y	L	S	Q	V	L	T	N	V	V	Y	F	F	V	V	R	Q
EPI_ISL_862039	Y	L	S	Q	V	L	T	N	V	V	Y	F	F	V	V	R	Q
_EPI_ISL_902737	Y	L	S	Q	V	L	T	N	V	V	Y	F	F	V	V	R	Q
_EPI_ISL_911707	Y	L	S	Q	V	L	T	N	V	V	Y	F	F	V	V	R	Q
_EPI_ISL_877131	Y	**F**	S	Q	V	L	T	N	V	V	Y	F	F	V	V	R	Q
_EPI_ISL_890185	Y	L	S	Q	V	L	**I**	N	V	V	Y	F	F	V	V	**H**	Q
_EPI_ISL_906050	Y	L	S	Q	V	L	T	N	V	V	Y	F	F	V	V	R	Q
EPI_ISL_576116	Y	L	S	Q	V	L	T	N	V	V	Y	F	F	V	**A**	R	Q
_EPI_ISL_877130	Y	**F**	S	Q	V	L	T	N	V	V	Y	F	F	V	V	R	Q
_EPI_ISL_877126	Y	**F**	S	Q	V	L	T	N	V	V	Y	F	F	V	V	R	Q
EPI_ISL_632936	Y	L	S	Q	V	L	T	N	V	V	Y	F	F	V	V	R	Q
_EPI_ISL_985398	Y	L	**F**	Q	V	L	T	N	V	V	Y	F	F	V	V	R	Q
_EPI_ISL_906052	Y	L	S	Q	V	L	T	N	V	V	Y	F	F	V	V	R	Q
_EPI_ISL_890187	Y	L	S	Q	V	L	T	N	V	V	Y	F	F	V	**A**	R	Q
_EPI_ISL_890186	Y	L	S	Q	V	L	T	N	V	V	Y	F	F	V	**A**	R	Q
EPI_ISL_632937	Y	L	S	Q	V	L	T	N	**L**	V	Y	F	F	V	V	R	Q
EPI_ISL_576115	Y	L	S	Q	V	L	T	N	V	V	Y	F	F	V	**A**	R	Q
_EPI_ISL_877127	Y	**F**	S	Q	V	L	T	N	V	V	Y	F	F	V	V	R	Q
_EPI_ISL_516800	Y	L	S	Q	V	L	T	N	V	V	Y	F	F	V	V	R	Q
EPI_ISL_1005697	Y	**F**	S	Q	V	L	T	N	V	V	Y	F	F	V	**A**	R	Q
EPI_ISL_576113	Y	L	S	Q	V	L	T	N	V	V	Y	F	F	V	**A**	R	Q
EPI_ISL_1005696	Y	L	S	Q	V	L	T	N	V	V	Y	F	F	V	**A**	R	Q
EPI_ISL_1005698	Y	L	S	Q	V	L	T	N	V	V	Y	F	F	V	**A**	R	Q
EPI_ISL_985397	Y	L	S	Q	V	**F**	T	N	V	V	Y	F	F	V	V	R	**K**
EPI_ISL_985396	Y	L	S	Q	V	L	T	N	V	V	Y	F	F	V	V	R	Q
EPI_ISL_525492	Y	L	S	Q	V	L	T	N	V	V	Y	F	F	V	V	R	Q
EPI_ISL_576145	Y	L	S	Q	V	L	T	N	V	V	Y	F	F	V	V	R	Q
EPI_ISL_906051	Y	L	S	Q	V	L	T	N	V	V	Y	F	F	V	V	R	Q
EPI_ISL_610161	Y	L	S	Q	V	L	T	N	V	V	Y	F	F	V	**A**	R	Q
EPI_ISL_516806	Y	L	S	Q	V	L	T	N	V	V	Y	F	F	V	V	R	Q
EPI_ISL_576128	Y	**X**	S	Q	V	L	T	N	V	V	Y	F	F	V	V	R	Q
EPI_ISL_576114	Y	L	S	Q	V	L	T	N	V	V	Y	F	F	V	V	R	Q
EPI_ISL_610162	Y	L	S	Q	V	L	T	N	V	V	Y	F	F	V	V	R	Q
EPI_ISL_516829	Y	L	S	Q	V	L	T	N	V	V	Y	F	F	V	V	R	Q
EPI_ISL_1005695	Y	L	S	Q	V	L	T	N	V	V	Y	F	F	**M**	V	R	Q
EPI_ISL_610155	Y	L	S	**K**	**A**	L	T	N	V	**I**	**D**	**C**	**V**	V	V	R	Q
EPI_ISL_610158	Y	L	S	Q	V	L	T	**Y**	V	V	Y	F	F	V	**E**	R	Q

	SPIKE GLYCOPROTEIN																
VIRUS ID	**254**	**258**	**371**	**390**	**394**	**397**	**400**	**494**	**570**	**614**	**675**	**677**	**679**	**689**	**808**	**811**	**832**
NC_045512.2 WUHAN	S	W	S	L	N	A	F	S	A	D	Q	Q	N	S	D	K	G
EPI_ISL_576130	S	W	S	L	N	A	F	S	A	**G**	Q	Q	N	S	D	K	G
_EPI_ISL_902749	S	W	S	L	N	A	F	S	A	**G**	Q	Q	N	S	D	K	G
_EPI_ISL_911709	S	W	S	L	N	A	F	**P**	A	**G**	Q	Q	N	**R**	D	K	G
EPI_ISL_885142	S	W	S	L	N	A	F	S	A	**G**	Q	Q	N	S	D	K	G
EPI_ISL_862040	S	W	S	L	N	A	F	S	A	**G**	Q	Q	N	S	D	K	G
_EPI_ISL_877129	S	W	S	L	N	A	F	S	A	**G**	Q	Q	N	S	D	K	G
_EPI_ISL_877128	S	W	S	L	N	A	F	S	A	**G**	Q	Q	N	S	D	K	G
EPI_ISL_862041	S	W	S	L	N	A	F	S	A	**G**	Q	Q	N	S	D	K	G
_EPI_ISL_891151	S	W	S	L	N	A	F	S	A	**G**	Q	Q	N	S	D	K	G
_EPI_ISL_905731	**F**	W	S	L	N	A	F	S	A	**G**	Q	Q	N	S	D	K	G
EPI_ISL_576383	S	W	S	L	N	A	F	S	A	**G**	Q	Q	N	S	D	K	G
EPI_ISL_872190	S	W	S	L	N	A	F	S	A	**G**	Q	Q	N	**R**	D	K	G
EPI_ISL_872189	S	W	S	L	N	A	F	S	A	**G**	**H**	Q	N	S	D	K	G
EPI_ISL_872188	S	W	S	L	N	A	F	S	A	**G**	Q	Q	N	S	D	K	G
EPI_ISL_575331	S	W	S	L	N	A	F	S	A	**G**	Q	Q	N	S	D	K	G
EPI_ISL_862039	S	W	S	L	N	A	F	S	A	**G**	Q	Q	N	S	D	K	G
_EPI_ISL_902737	S	W	S	L	N	A	F	S	A	**G**	Q	Q	N	S	D	K	G
_EPI_ISL_911707	S	W	S	L	N	A	F	**P**	A	**G**	Q	Q	N	**R**	D	K	G
_EPI_ISL_877131	S	W	S	L	N	A	F	S	A	**G**	Q	Q	N	S	D	K	G
_EPI_ISL_890185	S	W	S	L	N	A	F	S	A	**G**	Q	Q	N	S	D	K	G
_EPI_ISL_906050	S	W	S	L	N	A	F	S	**S**	**G**	Q	Q	**K**	S	D	K	G
EPI_ISL_576116	S	W	S	L	N	A	F	S	A	**G**	Q	Q	N	S	D	K	G
_EPI_ISL_877130	S	W	S	L	N	A	F	S	A	**G**	Q	Q	N	S	D	K	G
_EPI_ISL_877126	S	W	S	L	N	A	F	S	A	**G**	Q	Q	N	S	D	K	G
EPI_ISL_632936	S	W	S	L	N	A	F	S	A	**G**	Q	Q	N	S	D	**I**	G
_EPI_ISL_985398	S	W	S	L	N	A	F	S	A	**G**	Q	Q	N	**R**	D	K	G
_EPI_ISL_906052	S	W	S	L	N	A	F	S	**S**	**G**	Q	Q	**K**	S	D	K	G
_EPI_ISL_890187	S	W	S	L	N	A	F	S	A	**G**	Q	Q	N	S	D	K	G
_EPI_ISL_890186	S	W	S	L	N	A	F	S	A	**G**	Q	Q	N	S	D	K	G
EPI_ISL_632937	S	W	S	L	N	A	F	S	A	**G**	Q	**H**	N	S	D	K	G
EPI_ISL_576115	S	W	S	L	N	A	F	S	A	**G**	Q	Q	N	S	D	K	G
_EPI_ISL_877127	S	W	S	L	N	A	F	S	A	**G**	Q	Q	N	S	D	K	G
_EPI_ISL_516800	S	W	S	L	N	A	F	S	A	**G**	Q	Q	N	S	D	K	G
EPI_ISL_1005697	S	W	S	L	N	A	F	S	A	**G**	Q	Q	N	S	D	K	G
EPI_ISL_576113	S	W	S	L	N	A	F	S	A	**G**	Q	Q	N	S	D	K	G
EPI_ISL_1005696	S	W	S	L	N	A	F	S	A	**G**	Q	Q	N	S	D	K	G
EPI_ISL_1005698	S	W	S	L	N	A	F	S	A	**G**	Q	Q	N	S	D	K	G
EPI_ISL_985397	S	W	S	L	N	A	F	S	A	**G**	Q	Q	N	S	D	K	G
EPI_ISL_985396	S	W	S	L	N	A	F	S	A	**G**	Q	Q	N	S	D	K	G
EPI_ISL_525492	S	W	S	L	N	A	F	S	A	**G**	Q	Q	N	S	D	K	G
EPI_ISL_576145	S	W	S	L	N	A	F	S	A	**G**	Q	Q	N	S	D	K	G
EPI_ISL_906051	S	W	S	L	N	A	F	S	**S**	**G**	Q	Q	**K**	S	D	K	G
EPI_ISL_610161	S	W	S	L	N	A	F	S	A	**G**	Q	Q	N	S	D	K	G
EPI_ISL_516806	S	W	S	L	N	A	F	S	A	D	Q	Q	N	S	D	K	G
EPI_ISL_576128	S	W	S	L	N	A	F	S	A	**G**	Q	Q	N	S	D	K	G
EPI_ISL_576114	S	W	S	L	N	A	F	S	A	**G**	Q	Q	N	S	D	K	G
EPI_ISL_610162	S	**R**	S	L	N	A	F	S	A	**G**	Q	Q	N	S	D	K	G
EPI_ISL_516829	S	W	S	L	N	A	F	S	A	**G**	Q	Q	N	S	D	K	G
EPI_ISL_1005695	S	W	S	L	N	A	F	S	A	**G**	Q	Q	N	S	D	K	**D**
EPI_ISL_610155	S	W	**P**	**I**	**K**	**T**	**L**	S	A	**G**	Q	Q	N	S	**E**	K	G
EPI_ISL_610158	S	W	S	L	N	A	F	S	A	D	Q	Q	N	S	D	K	G

	SPIKE GLYCOPROTEIN						**NS3-ORF3A**										
VIRUS ID	**954**	**958**	**959**	**960**	**962**	**1259**	**21**	**54**	**57**	**66**	**67**	**71**	**99**	**104**	**106**	**144**	**151**
NC_045512.2 WUHAN	Q	A	L	N	L	D	K	A	Q	K	K	L	A	P	L	N	T
EPI_ISL_576130	Q	A	L	N	L	D	K	A	**H**	K	K	L	A	P	L	N	T
_EPI_ISL_902749	Q	A	L	N	L	D	**N**	A	**H**	K	K	L	A	P	L	N	T
_EPI_ISL_911709	Q	A	L	N	L	D	K	A	**H**	K	K	L	A	P	L	N	T
EPI_ISL_885142	Q	A	L	N	L	D	K	A	**H**	K	K	L	A	P	L	N	T
EPI_ISL_862040	Q	A	L	N	L	D	K	A	Q	K	K	L	A	P	L	N	T
_EPI_ISL_877129	Q	A	L	N	L	D	K	A	**H**	**N**	K	L	A	P	L	N	T
_EPI_ISL_877128	Q	A	L	N	L	D	K	A	**H**	**N**	K	L	A	P	L	N	T
EPI_ISL_862041	Q	A	L	N	L	D	K	A	Q	K	K	L	A	P	L	**S**	T
_EPI_ISL_891151	Q	A	L	N	L	D	K	A	**H**	K	**N**	L	A	P	L	N	T
_EPI_ISL_905731	Q	A	L	N	L	D	**N**	A	**H**	K	K	L	A	P	L	N	T
EPI_ISL_576383	Q	A	L	N	L	D	K	A	Q	K	K	L	A	P	L	N	T
EPI_ISL_872190	Q	A	L	N	L	D	K	A	**H**	K	K	L	A	P	L	N	T
EPI_ISL_872189	Q	A	L	N	L	D	K	A	**H**	K	K	L	A	P	L	N	T
EPI_ISL_872188	Q	A	L	N	L	D	K	A	**H**	K	K	L	A	P	L	N	T
EPI_ISL_575331	Q	A	L	N	L	D	K	A	**H**	K	K	L	A	P	L	N	T
EPI_ISL_862039	Q	A	L	N	L	D	K	A	**H**	K	K	L	A	P	L	N	T
_EPI_ISL_902737	Q	A	L	N	L	D	**N**	A	**H**	K	K	L	A	P	L	N	T
_EPI_ISL_911707	Q	A	L	N	L	D	K	A	**H**	K	K	L	A	P	L	N	T
_EPI_ISL_877131	Q	A	L	N	L	D	K	A	**H**	**N**	K	L	A	P	L	N	T
_EPI_ISL_890185	Q	A	L	N	L	D	K	A	Q	K	K	L	A	P	L	N	T
_EPI_ISL_906050	Q	A	L	N	L	**Y**	K	A	**H**	K	K	L	A	P	L	N	T
EPI_ISL_576116	Q	A	L	N	L	D	K	A	**H**	K	K	L	A	P	L	N	T
_EPI_ISL_877130	Q	A	L	N	L	D	K	A	**H**	**N**	K	L	A	P	L	N	T
_EPI_ISL_877126	Q	A	L	N	L	D	K	A	**H**	**N**	K	L	A	P	L	N	T
EPI_ISL_632936	Q	A	L	N	L	D	K	A	**H**	K	K	L	A	P	L	N	**I**
_EPI_ISL_985398	Q	A	L	N	L	D	K	A	**H**	K	K	L	A	**T**	L	N	T
_EPI_ISL_906052	Q	A	L	N	L	**Y**	K	A	**H**	K	K	L	A	P	L	N	T
_EPI_ISL_890187	Q	A	L	N	L	D	K	A	**H**	K	K	L	A	P	L	N	T
_EPI_ISL_890186	Q	A	L	N	L	D	K	A	**H**	K	K	L	A	P	L	N	T
EPI_ISL_632937	Q	A	L	N	L	D	K	A	**H**	K	K	L	A	P	L	N	T
EPI_ISL_576115	Q	A	L	N	L	D	K	A	**H**	K	K	L	A	P	L	N	T
_EPI_ISL_877127	Q	A	L	N	L	D	K	A	**H**	**N**	K	L	A	P	L	N	T
_EPI_ISL_516800	Q	A	L	N	L	D	K	A	**H**	K	K	L	A	P	L	N	T
EPI_ISL_1005697	Q	A	L	N	L	D	K	A	**H**	K	K	L	A	P	**F**	N	T
EPI_ISL_576113	Q	A	L	N	L	D	K	A	**H**	K	K	L	A	P	L	N	T
EPI_ISL_1005696	Q	A	L	N	L	D	K	A	**H**	K	K	L	A	P	L	N	T
EPI_ISL_1005698	Q	A	L	N	L	D	K	A	**H**	K	K	L	A	P	L	N	T
EPI_ISL_985397	Q	A	L	N	L	D	K	A	**H**	K	K	L	A	P	L	N	T
EPI_ISL_985396	Q	A	L	N	L	D	K	A	**H**	K	K	L	A	P	L	N	T
EPI_ISL_525492	Q	A	L	N	L	D	K	A	**H**	K	K	L	A	P	L	N	T
EPI_ISL_576145	Q	A	L	N	L	D	K	A	Q	K	K	L	A	P	L	N	T
EPI_ISL_906051	Q	A	L	N	L	**Y**	K	A	**H**	K	K	L	A	P	L	N	T
EPI_ISL_610161	Q	A	L	N	L	D	K	A	**H**	K	K	L	A	P	L	N	T
EPI_ISL_516806	Q	A	L	N	L	D	K	A	Q	K	K	L	A	P	L	N	T
EPI_ISL_576128	Q	A	L	N	L	D	K	A	**H**	K	K	L	A	P	L	N	T
EPI_ISL_576114	Q	A	L	N	L	D	K	A	**H**	K	K	L	A	P	L	N	T
EPI_ISL_610162	Q	A	L	N	L	D	K	A	Q	K	K	L	A	P	L	N	T
EPI_ISL_516829	Q	A	L	N	L	D	K	**V**	**H**	K	K	L	**S**	P	L	N	T
EPI_ISL_1005695	Q	A	L	N	L	D	K	A	Q	K	K	L	A	P	L	N	T
EPI_ISL_610155	**K**	**T**	*	**K**	**H**	D	K	A	**H**	K	K	L	A	P	L	N	T
EPI_ISL_610158	Q	A	L	N	L	D	K	A	**H**	K	K	**R**	A	P	L	N	T

	NS3-ORF3A									**MEMBRANE GLYCOPROTEIN**							**ORF7A**
VIRUS ID	**171**	**202**	**207**	**222**	**223**	**224**	**260**	**262**	**275**	**2**	**7**	**8**	**35**	**37**	**210**	**212**	**47**
NC_045512.2 WUHAN	S	V	F	D	T	G	M	P	N	A	T	I	L	F	H	S	H
EPI_ISL_576130	S	V	F	D	T	G	M	P	N	A	T	I	L	F	H	S	H
_EPI_ISL_902749	S	V	F	D	T	G	M	**L**	N	**S**	T	I	L	F	H	S	**N**
_EPI_ISL_911709	S	V	F	D	T	G	M	P	N	A	T	I	L	F	H	S	H
EPI_ISL_885142	S	V	F	D	T	**C**	M	P	N	A	T	I	L	F	H	S	H
EPI_ISL_862040	S	V	F	D	T	G	M	P	N	A	T	I	L	F	H	S	H
_EPI_ISL_877129	**L**	V	F	D	T	G	M	P	N	A	T	I	L	F	H	S	H
_EPI_ISL_877128	**L**	V	F	D	T	G	M	P	N	A	T	I	L	F	H	S	H
EPI_ISL_862041	S	V	F	D	T	G	M	P	N	A	T	I	L	F	H	S	H
_EPI_ISL_891151	S	V	F	D	T	G	M	P	N	A	T	I	L	F	H	S	H
_EPI_ISL_905731	S	V	F	D	T	G	M	**L**	N	**S**	T	I	L	F	H	S	H
EPI_ISL_576383	S	V	F	D	T	G	M	P	N	A	T	I	L	F	H	S	H
EPI_ISL_872190	S	V	F	D	T	G	M	P	N	A	T	I	L	F	H	S	H
EPI_ISL_872189	S	V	F	D	**I**	G	**I**	P	**E**	A	T	I	L	F	H	S	H
EPI_ISL_872188	S	V	F	D	T	G	M	P	N	A	T	I	L	F	H	S	H
EPI_ISL_575331	S	V	F	D	T	G	M	P	N	A	T	I	L	F	H	S	H
EPI_ISL_862039	S	V	F	D	T	**C**	M	P	N	A	T	I	L	F	H	S	H
_EPI_ISL_902737	S	V	F	D	T	G	M	**L**	N	**S**	T	I	L	F	H	S	H
_EPI_ISL_911707	S	V	F	D	T	G	M	P	N	A	T	I	L	F	H	S	H
_EPI_ISL_877131	**L**	V	F	D	T	G	M	P	N	A	T	I	L	F	H	S	H
_EPI_ISL_890185	**L**	V	F	D	T	G	M	P	N	A	T	I	L	F	H	S	H
_EPI_ISL_906050	S	V	F	D	T	G	M	P	N	A	T	I	L	F	H	S	H
EPI_ISL_576116	S	V	F	D	T	G	M	P	N	A	T	I	L	F	H	S	H
_EPI_ISL_877130	**L**	V	F	D	T	G	M	P	N	A	T	I	L	F	H	S	H
_EPI_ISL_877126	**L**	V	F	D	T	G	M	P	N	A	T	I	L	F	H	S	H
EPI_ISL_632936	S	V	F	D	T	G	M	P	N	A	T	I	L	F	H	S	H
_EPI_ISL_985398	S	**L**	F	D	T	G	M	P	N	A	T	I	L	F	H	S	H
_EPI_ISL_906052	S	V	F	D	T	G	M	P	N	A	T	I	L	F	H	S	H
_EPI_ISL_890187	S	V	F	D	T	G	M	P	N	A	T	I	L	F	H	S	H
_EPI_ISL_890186	S	V	F	D	T	G	M	P	N	A	T	I	L	F	H	S	H
EPI_ISL_632937	S	V	F	**Y**	T	G	M	P	N	A	T	I	L	F	H	S	H
EPI_ISL_576115	S	V	F	D	T	G	M	P	N	A	T	I	L	F	H	S	H
_EPI_ISL_877127	**L**	V	F	D	T	G	M	P	N	A	T	I	L	F	H	S	H
_EPI_ISL_516800	S	V	F	D	T	G	M	P	N	A	T	I	L	F	H	S	H
EPI_ISL_1005697	S	V	F	D	T	G	M	P	N	A	T	I	L	F	H	S	H
EPI_ISL_576113	S	V	F	D	T	G	M	P	N	A	T	I	L	F	H	S	H
EPI_ISL_1005696	S	V	F	D	T	G	M	P	N	A	T	I	L	F	H	S	H
EPI_ISL_1005698	S	V	F	D	T	G	M	P	N	A	T	I	L	F	H	S	H
EPI_ISL_985397	S	V	F	D	T	G	M	P	N	A	T	I	L	F	H	S	H
EPI_ISL_985396	S	V	F	D	T	G	M	P	N	A	T	I	L	F	H	S	H
EPI_ISL_525492	S	V	F	D	T	G	M	P	N	A	T	I	L	F	H	S	H
EPI_ISL_576145	S	V	F	D	T	G	M	P	N	A	T	I	L	F	H	S	H
EPI_ISL_906051	S	V	F	D	T	G	M	P	N	A	T	I	L	F	H	S	H
EPI_ISL_610161	S	V	F	D	T	G	M	P	N	A	T	I	L	F	H	S	H
EPI_ISL_516806	S	V	F	D	T	G	M	P	N	A	T	I	L	F	H	S	H
EPI_ISL_576128	S	V	F	D	T	G	M	P	N	A	T	I	L	F	H	S	H
EPI_ISL_576114	S	V	F	D	T	G	M	P	N	A	T	I	L	F	H	S	H
EPI_ISL_610162	S	V	F	D	T	G	M	P	N	A	T	I	L	F	H	S	H
EPI_ISL_516829	S	V	F	D	T	G	M	P	N	A	T	I	L	F	H	S	H
EPI_ISL_1005695	S	V	F	D	T	G	M	P	N	A	T	I	L	F	H	S	H
EPI_ISL_610155	S	V	**I**	D	T	G	M	P	N	A	**N**	**S**	**R**	**L**	H	S	H
EPI_ISL_610158	S	V	F	D	T	G	M	P	N	A	T	I	L	F	**Q**	**C**	H

	**ORF7A**		**ORF7B**		**ORF8**				**NP-N**								
VIRUS ID	**73**	**116**	**13**	**19**	**11**	**43**	**62**	**65**	**13**	**101**	**103**	**119**	**128**	**151**	**155**	**160**	**193**
NC_045512.2 WUHAN	H	L	F	F	T	S	V	A	P	M	D	A	D	P	A	Q	S
EPI_ISL_576130	H	L	F	F	T	S	V	A	P	M	D	A	D	P	A	Q	S
_EPI_ISL_902749	H	L	F	F	T	S	**L**	A	P	M	**Y**	A	D	P	A	Q	S
_EPI_ISL_911709	H	L	F	F	T	S	V	A	P	M	D	**S**	D	P	A	Q	S
EPI_ISL_885142	H	L	F	F	T	S	V	A	P	M	D	A	D	P	A	Q	S
EPI_ISL_862040	H	L	**L**	F	T	**V**	V	A	P	M	D	A	D	**S**	A	Q	S
_EPI_ISL_877129	H	L	F	F	T	S	V	A	P	M	D	A	**Y**	P	A	Q	S
_EPI_ISL_877128	H	L	F	F	T	S	V	A	P	M	D	A	**Y**	P	A	Q	S
EPI_ISL_862041	H	L	F	F	**I**	S	V	A	P	M	D	A	D	P	A	Q	S
_EPI_ISL_891151	H	L	F	F	T	S	V	**S**	P	M	D	A	D	P	A	Q	S
_EPI_ISL_905731	H	L	F	F	T	S	**L**	A	P	M	**Y**	A	D	P	A	Q	S
EPI_ISL_576383	H	L	F	F	T	S	V	A	P	M	D	A	D	P	A	Q	S
EPI_ISL_872190	H	L	F	**S**	T	S	V	A	P	M	D	**S**	D	P	A	Q	S
EPI_ISL_872189	H	L	F	F	T	S	V	A	P	M	D	A	D	P	A	Q	S
EPI_ISL_872188	H	L	F	F	T	S	V	A	P	M	D	A	D	P	A	Q	S
EPI_ISL_575331	H	L	F	F	T	S	V	A	P	M	D	A	D	P	A	Q	S
EPI_ISL_862039	H	L	F	F	T	S	V	A	P	M	D	A	D	P	A	Q	S
_EPI_ISL_902737	H	L	F	F	T	S	**L**	A	P	M	**Y**	A	D	P	A	Q	S
_EPI_ISL_911707	H	L	F	F	T	S	V	A	P	M	D	**S**	D	P	A	Q	S
_EPI_ISL_877131	H	L	F	F	T	S	V	A	P	M	D	A	**Y**	P	A	Q	S
_EPI_ISL_890185	H	L	F	F	T	S	V	A	P	M	D	A	D	P	**V**	Q	S
_EPI_ISL_906050	H	L	F	F	T	S	V	A	P	M	D	A	D	P	A	Q	S
EPI_ISL_576116	H	L	F	F	T	S	V	A	P	M	D	**S**	D	P	A	Q	**I**
_EPI_ISL_877130	H	L	F	F	T	S	V	A	P	M	D	A	**Y**	P	A	Q	S
_EPI_ISL_877126	H	L	F	F	T	S	V	A	P	M	D	A	**Y**	P	A	Q	S
EPI_ISL_632936	H	L	F	F	T	S	V	A	P	M	D	A	D	P	A	Q	S
_EPI_ISL_985398	H	L	F	F	T	S	V	A	P	**I**	D	**S**	D	P	A	Q	S
_EPI_ISL_906052	H	L	F	F	T	S	V	A	P	M	D	A	D	P	A	Q	S
_EPI_ISL_890187	H	L	F	F	T	S	V	A	P	M	D	**S**	D	P	A	Q	**I**
_EPI_ISL_890186	H	L	F	F	T	S	V	A	P	M	D	**S**	D	P	A	Q	**I**
EPI_ISL_632937	H	L	F	F	T	S	V	A	P	M	D	A	D	P	A	Q	S
EPI_ISL_576115	H	L	F	F	T	S	V	A	P	M	D	**S**	D	P	A	Q	**I**
_EPI_ISL_877127	H	L	F	F	T	S	V	A	P	M	D	A	**Y**	P	A	Q	S
_EPI_ISL_516800	H	L	F	F	T	S	V	A	P	M	D	A	D	P	A	Q	S
EPI_ISL_1005697	H	L	F	F	T	S	V	A	P	M	D	**S**	D	P	A	Q	**I**
EPI_ISL_576113	H	L	F	F	T	S	V	A	P	M	D	**S**	D	P	A	Q	**I**
EPI_ISL_1005696	H	L	F	F	T	S	V	A	P	M	D	**S**	D	P	A	Q	**I**
EPI_ISL_1005698	H	L	F	F	T	S	V	A	P	M	D	**S**	D	P	A	Q	**I**
EPI_ISL_985397	H	L	F	F	T	S	V	A	P	M	D	A	D	P	A	Q	S
EPI_ISL_985396	H	L	F	F	T	S	V	A	P	M	D	A	D	P	A	Q	**I**
EPI_ISL_525492	**Y**	L	F	F	T	S	V	A	P	M	D	A	D	P	A	Q	S
EPI_ISL_576145	H	L	F	F	T	S	V	A	P	M	D	A	D	P	A	Q	S
EPI_ISL_906051	H	L	F	F	T	S	V	A	P	M	D	A	D	P	A	Q	S
EPI_ISL_610161	H	L	F	F	T	S	V	A	P	M	D	**S**	D	P	A	Q	**I**
EPI_ISL_516806	H	L	F	F	T	S	V	A	P	M	D	A	D	P	A	Q	S
EPI_ISL_576128	H	L	F	F	T	S	V	A	P	M	D	A	D	P	A	Q	S
EPI_ISL_576114	H	L	F	F	T	S	V	A	P	M	D	A	D	P	A	Q	S
EPI_ISL_610162	H	L	F	F	T	S	V	A	**L**	M	D	A	D	P	A	Q	S
EPI_ISL_516829	H	L	F	F	T	S	V	A	P	M	D	A	D	P	A	**R**	S
EPI_ISL_1005695	H	L	F	F	T	S	V	A	P	M	D	A	D	P	A	Q	S
EPI_ISL_610155	H	**P**	F	F	T	S	V	A	P	M	D	A	D	P	A	Q	S
EPI_ISL_610158	H	L	F	F	T	S	V	A	P	M	D	A	D	P	A	Q	S

	**NP-N**								**ORF10**		**3'UTR**						
VIRUS ID	**194**	**195**	**199**	**203**	**204**	**205**	**234**	**235**	**10**	**29**	**11**	**30**	**32**	**61**	**64**	**65**	**66**
NC_045512.2 WUHAN	S	R	P	R	G	T/G	M	S	P	Q	G	Q	T	F	E	*	Q
EPI_ISL_576130	S	R	P	R	G	T	M	S	P	Q	G	Q	T	F	E	*	Q
_EPI_ISL_902749	S	R	P	R	G	**I**	M	S	P	Q	G	Q	T	F	E	*	Q
_EPI_ISL_911709	S	R	P	R	G	T	**I**	S	P	Q	G	Q	T	F	E	*	Q
EPI_ISL_885142	S	R	P	R	G	**I**	M	S	P	Q	**V**	Q	T	**C**	E	*	Q
EPI_ISL_862040	S	R	P	**K**	G	**-**	M	S	P	Q	G	Q	T	F	E	*	Q
_EPI_ISL_877129	S	R	P	R	G	T	M	**F**	P	Q	G	Q	T	F	E	*	Q
_EPI_ISL_877128	S	R	P	R	G	T	M	**F**	P	Q	G	Q	T	F	E	*	Q
EPI_ISL_862041	S	R	P	**K**	G	**-**	M	S	P	Q	G	Q	T	F	E	*	Q
_EPI_ISL_891151	**L**	R	P	R	G	T	M	S	P	Q	G	Q	T	F	E	*	**K**
_EPI_ISL_905731	S	R	P	R	G	**I**	M	S	P	Q	G	Q	T	F	E	*	Q
EPI_ISL_576383	S	R	**S**	**K**	G	**-**	M	S	P	Q	G	Q	T	F	E	**R**	**K**
EPI_ISL_872190	S	R	P	R	G	T	M	S	P	Q	G	Q	**I**	F	E	*	Q
EPI_ISL_872189	S	R	P	R	G	T	**I**	S	P	Q	G	Q	T	F	E	*	Q
EPI_ISL_872188	**L**	R	P	R	G	T	M	S	P	Q	G	*	T	F	E	*	Q
EPI_ISL_575331	S	R	P	R	G	T	M	S	P	Q	G	Q	T	F	E	*	Q
EPI_ISL_862039	S	R	P	R	G	**I**	M	S	P	Q	**V**	Q	T	**C**	E	*	Q
_EPI_ISL_902737	S	R	P	R	G	**I**	M	S	P	Q	G	Q	T	F	E	*	Q
_EPI_ISL_911707	S	R	P	R	G	T	**I**	S	P	Q	G	Q	T	F	E	*	Q
_EPI_ISL_877131	S	R	P	R	G	T	M	**F**	P	Q	G	Q	T	F	E	*	Q
_EPI_ISL_890185	S	R	P	**K**	G	**-**	M	S	P	Q	**V**	Q	T	F	E	*	Q
_EPI_ISL_906050	S	R	P	R	G	T	M	**F**	P	Q	G	Q	T	F	E	*	**K**
EPI_ISL_576116	S	R	P	R	G	T	M	S	P	Q	G	Q	T	F	**X**	*	Q
_EPI_ISL_877130	S	R	P	R	G	T	M	**F**	P	Q	G	Q	T	F	E	*	Q
_EPI_ISL_877126	S	R	P	R	G	T	M	**F**	P	Q	G	Q	T	F	E	*	Q
EPI_ISL_632936	S	R	P	R	G	T	M	S	P	Q	G	Q	T	F	E	*	Q
_EPI_ISL_985398	S	R	P	R	G	T	M	S	P	Q	G	Q	T	F	E	*	Q
_EPI_ISL_906052	S	R	P	R	G	T	M	**F**	P	Q	G	Q	T	F	E	*	**K**
_EPI_ISL_890187	S	R	P	R	G	T	M	S	P	Q	G	Q	T	F	E	*	Q
_EPI_ISL_890186	S	R	P	R	G	T	M	S	P	Q	G	Q	T	F	E	*	Q
EPI_ISL_632937	S	R	P	R	G	T	**I**	S	P	*	G	Q	T	F	E	*	**K**
EPI_ISL_576115	S	R	P	R	G	T	M	S	P	Q	G	Q	T	F	E	*	Q
_EPI_ISL_877127	S	R	P	R	G	T	M	**F**	P	Q	G	Q	T	F	E	*	Q
_EPI_ISL_516800	S	R	P	R	G	T	M	S	P	Q	G	Q	T	F	E	*	Q
EPI_ISL_1005697	S	R	P	R	G	T	M	S	P	Q	G	Q	T	F	E	*	Q
EPI_ISL_576113	S	R	P	R	G	T	M	S	P	Q	G	Q	T	F	E	*	Q
EPI_ISL_1005696	S	R	P	R	G	T	M	S	P	Q	G	Q	T	F	E	*	Q
EPI_ISL_1005698	S	R	P	R	G	T	M	S	P	Q	G	Q	T	F	E	*	Q
EPI_ISL_985397	S	R	P	R	G	T	M	S	P	Q	G	Q	T	F	E	*	Q
EPI_ISL_985396	S	R	P	R	G	T	M	S	P	Q	G	Q	T	F	E	*	Q
EPI_ISL_525492	S	R	P	R	G	T	M	S	P	Q	G	Q	T	F	E	*	Q
EPI_ISL_576145	S	**S**	P	**K**	**R**	T	M	S	P	Q	G	Q	T	F	E	*	Q
EPI_ISL_906051	S	R	P	R	G	T	M	**F**	P	Q	G	Q	T	F	E	*	Q
EPI_ISL_610161	S	R	P	R	G	T	M	S	P	Q	G	Q	T	F	E	*	Q
EPI_ISL_516806	S	R	P	R	G	T	M	S	P	Q	G	Q	T	F	E	*	Q
EPI_ISL_576128	S	R	P	R	G	T	M	S	P	Q	G	Q	T	F	E	*	Q
EPI_ISL_576114	S	R	P	R	G	T	M	S	P	Q	G	Q	T	F	E	*	Q
EPI_ISL_610162	S	R	P	R	G	T	M	S	P	Q	G	Q	T	F	E	*	Q
EPI_ISL_516829	S	R	P	R	G	T	M	S	P	Q	G	Q	T	F	E	*	Q
EPI_ISL_1005695	S	R	P	R	G	T	M	S	P	Q	G	Q	T	F	E	*	Q
EPI_ISL_610155	S	R	P	R	G	T	M	S	P	Q	G	Q	T	F	E	*	Q
EPI_ISL_610158	S	R	P	R	G	T	M	S	**S**	Q	G	Q	T	F	E	*	Q

**Figure 1:**
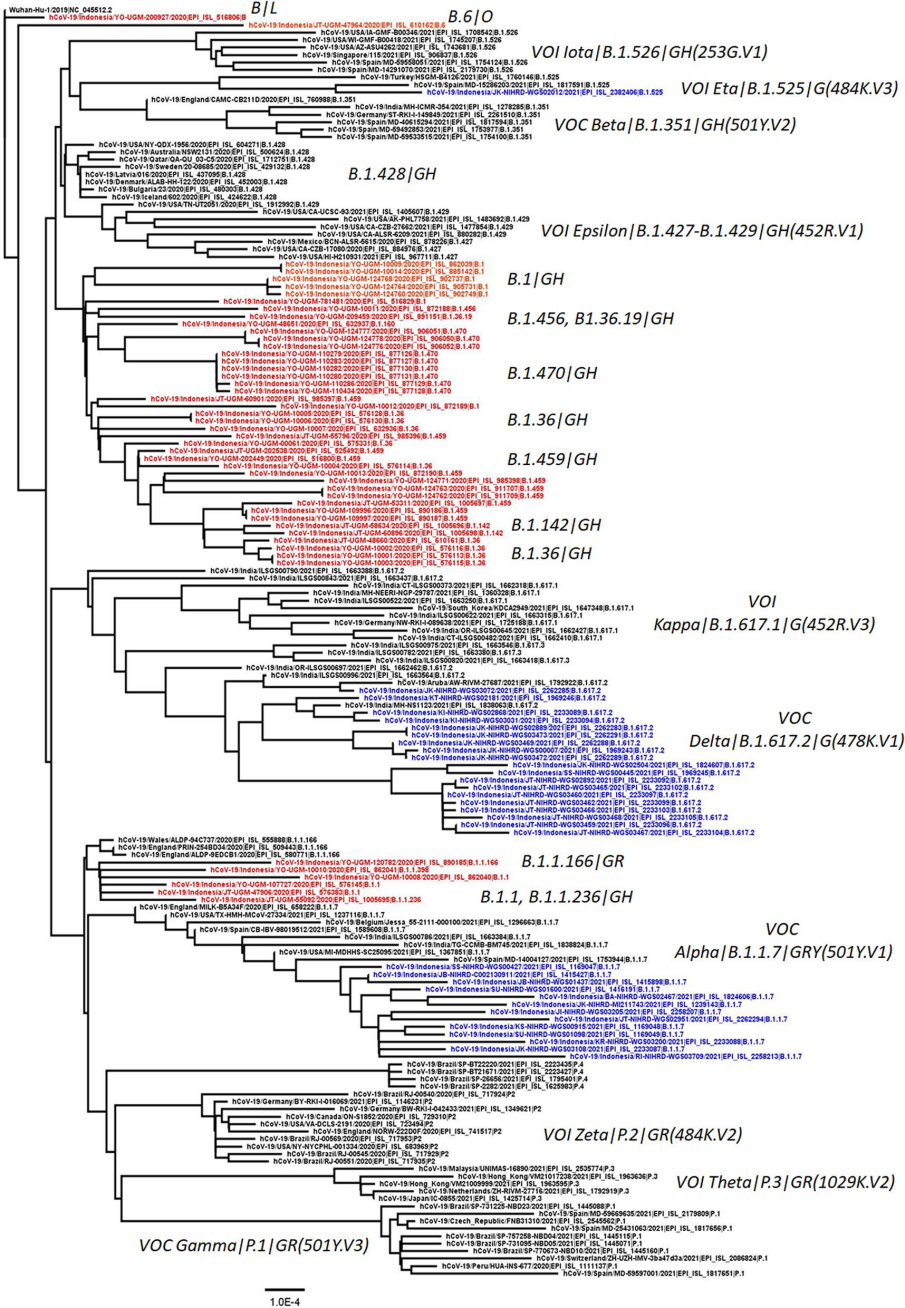
The evolutionary history was inferred using the Neighbor-Joining method^11^. The optimal tree is shown. The percentage of replicate trees in which associated taxa clustered together in the bootstrap test (1000 replicates) are shown next to the branches^12^. The tree is drawn to scale, with branch lengths in the same units as those of the evolutionary distances used to infer the phylogenetic tree. The evolutionary distances were computed using the Kimura 2-parameter method^13^ and are in the units of the number of base substitutions per site. This analysis involved 170 nucleotide sequences. All ambiguous positions were removed for each sequence pair (pairwise deletion option). There was a total of 29,563 positions in the final dataset. Evolutionary analyses were conducted in MEGA10.

Phylogenetic analysis showed that although most virus samples belonged to the clade GH and followed by the clade GR, none of these was detected as the variant of concern (VOC) and the variant of interest (VOI) of SARS-CoV-2 (Fig. 1). While two viruses of clade L and O formed separate clusters to the GH and GR clades.

### Association between multiple S protein mutations with COVID-19 patients’ outcomes and prognostic factors

There was no significant association between multiple S protein mutations with either hospitalization or mortality of COVID-19 patients (*p* = 0.11 and 0.69, respectively) (Table 4). Moreover, none of the prognostic factors was associated with multiple S protein mutations (*p* > 0.05) (Table 4).

**Table 4:** Association between multiple S protein mutations with outcomes of patients with COVID-19 and prognostic factors.

Variables	S protein mutation		OR (95% CI)	*p*-value
	Multiple (n, %; mean ± SD)	None/single (n, %; mean ± SD)		
**Hospitalized**				
Yes	16 (48.5)	13 (72.2)	0.36 (0.11–1.25)	0.11
No	17 (51.5)	5 (27.8)		
**Survival**				
Died	5 (15.2)	2 (11.1)	1.43 (0.25–8.23)	0.69
Live	28 (84.8)	16 (88.9)		
RT-PCR Ct value	19.2 ± 3.7	20.6 ± 4.7		0.26
**Age (years)**				
≥ 65	7 (21.2)	3 (16.7)	1.35 (0.30–6.0)	0.70
< 65	26 (78.8)	15 (83.3)		
**Sex**				
Male	19 (57.6)	11 (61.1)	0.86 (0.27–2.79)	0.81
Female	14 (42.4)	7 (38.9)		
**Comorbidity**				
Obesity	2 (6.1)	1 (5.6)	1.10 (0.09–13.0)	0.94
Diabetes	11 (33.3)	4 (22.2)	1.75 (0.46–6.59)	0.41
Hypertension	6 (18.2)	8 (44.4)	0.28 (0.08–1.0)	0.05
Cardiovascular disease	6 (18.2)	3 (16.7)	1.11 (0.24–5.09)	0.89
Chronic kidney disease	2 (6.1)	0	2.94 (0.14–64.55)	0.49
Smoking	2 (6.1)	2 (11.1)	0.51 (0.07–4.01)	0.53
**Therapy**				
ACEI/ARB	3 (9.1)	1 (5.6)	1.7 (0.16–17.65)	0.66
Anticoagulant	7 (21.2)	4 (22.2)	0.94 (0.23–3.78)	0.93
Steroid	4 (12.1)	1 (5.6)	2.34 (0.24–22.73)	0.46

*Significant.

*ACEI* angiotensin-converting enzyme inhibitors, *ARB* angiotensin receptor blocker, *CI* confidence interval, *OR* odds ratio.

### Multivariate analysis

Multivariate analysis showed that hypertension and anticoagulant therapy were the substantial factors affecting the hospitalization and mortality of patients with COVID-19 with a *p*-value of 0.009 (OR = 17.06 [95% CI 2.02–144.36]) and 0.001 (OR = 46.8 (95% CI 4.63–472.77), respectively. Interestingly, the multiple S protein mutations almost reached a significant level affecting the hospitalization of patients (*p* = 0.07) with the OR of 4.64 (95% CI 0.87–24.68) (Table 5).

**Table 5:** Multivariate analysis of the association between prognostic factors and outcomes of patients with COVID-19.

Prognostic factor	Hospitalized		Mortality	
	OR (95% CI)	*p*-value	OR (95% CI)	*p*-value
Multiple S protein mutations	4.64 (0.87–24.68)	0.07	0.91 (0.04–22.85)	0.96
Age (≥ 65 years)	0.10 (0.004–3.07)	0.19	4.56 (0.01–2267.77)	0.63
Sex (male)	2.5 (0.5–12.6)	0.24	3.45 (0.01–941.16)	0.67
**Comorbidity**				
Obesity	–	1	0.05 (0.0001–23.23)	0.33
Diabetes	2.74 (0.33–22.76)	0.35	14.27 (0.16–1286.04)	0.25
Hypertension	17.06 (2.02–144.36)	0.009	3.44 (0.03–386.31)	0.61
Cardiovascular disease	5.52 (0.18–168.92)	0.33	1.31 (0.01–340.36)	0.92
Chronic kidney disease	–	1	3.68 (0.04–353.38)	0.58
Smoking	6.72 (0.23–197.53)	0.27	–	1
**Therapy**				
ACEI/ARB	–	1	13.69 (0.02–11,919.51)	0.45
Anticoagulant	–	1	46.8 (4.63–472.77)	0.001*
Steroid	–	1	43.96 (0.05–41,926.76)	0.28

*Significant (*p* < 0.05).

*ACEI* angiotensin-converting enzyme inhibitors, *ARB* angiotensin receptor blocker, *CI* confidence interval, *OR* odds ratio.

## Discussion

Our first study in Indonesia investigates the association between several prognostic factors and disease outcomes of COVID-19 patients infected with SARS-CoV-2 harboring multiple S protein mutations. Indeed, our findings showed the effect of hypertension and anticoagulants on the severity of COVID-19 patients from the Indonesian population. Based on our study, the patients with hypertension have a ~ 17-fold higher risk of hospitalization than those without hypertension, in line with previous reports^11,19^. In a small retrospective study in China examining 191 patients of the early pandemic, the percentage of patients with hypertension was significantly higher in the non-survivor group than the survivor group (48% vs. 23%, respectively)^20^. The association between hypertension and increased risk of severe COVID-19 was confirmed by a meta-analysis study with a total of 2,893 patients. The study found that hypertension was associated with about a 2.5-fold increase of severe and fatal COVID-19 cases^21^.

The pathogenesis of hypertension affecting the COVID-19 severity is complex. Thus, the effect of hypertension on COVID-19 severity is controversial^22^. Indeed, a more extensive population study in England involving more than 17 million health records showed no association between hypertension and COVID-19 mortality after total adjustment^23^. Noteworthy, the impact of hypertension on the severity of COVID-19 is significant when accompanied by cardiovascular diseases, including myocardial injury^24^. However, our study did not show an association between the use of angiotensin-converting enzyme inhibitors/angiotensin receptor blockers (ACEI/ARB) and COVID-19 severity. Similar to hypertension, the effect of ACEI/ARB on COVID-19 severity is still inconclusive^23^. The S protein of SARS-CoV-2 binds to the ACE2 receptor to enter the human cells, suggesting that the use of ACEI/ARB might worsen the prognosis of COVID-19^25^. The downregulation of ACE2 resulted in the upregulation of interleukin 6, one of the pivotal mediators of cytokines storm in severe COVID-19 patients^26^. However, current reports showed that ACEI/ARB was not associated with the poorer outcomes of COVID-19 patients^27,28^.

Our study also demonstrated the association between anticoagulant therapy and COVID-19 mortality with an increased risk of approximately ~ 47-fold. SARS-CoV-2 often induces a pro-coagulative state due to several mechanisms, including endothelial dysfunction, cytokine storm, and complement hyperactivation^29^. While a recent study showed that anticoagulant therapies decreased the mortality of patients with COVID-19, it was not the case with our findings. These differences might be because we grouped the hospitalized and non-hospitalized into one group, classified into anticoagulant versus non-anticoagulant groups. Of note, we have only a limited sample size. These limitations should be considered during the interpretation of our findings. Further study with larger sample size is necessary to clarify and confirm our study.

Most previous reports focused on the impact of VOC on the COVID-19 outcomes, including B.1.1.7 (alpha), B.1.351 (beta), P1 (gamma), and the most recent VOC, B.1.617.2 (delta)^30–34^. Indonesia has reported identifying alpha, beta, and delta variants since January 2021^35^. In this present study, we have not found the VOC and VOI strains in our samples collected from June to October 2020 or before the first detection of VOC (B.1.1.7 lineage) in Indonesia in January 2021. Currently, the delta variant is identified as the most frequent VOC^35^. However, the actual frequency of the circulating VOCs in Indonesia might be biased due to our limited whole-genome sequencing capacities.

Interestingly, we revealed that patients with multiple S protein mutations might have a ~ fivefold higher possibility of being hospitalized than those with none or a single S protein mutation. However, the association between mutation and clinical outcome of COVID-19 is inconclusive. A study in Uruguay found that mutation in structural and non-structural protein was not associated with COVID-19 fatalities^36^. Another recent study analyzed the association between viral genomic variants and COVID-19 outcomes. They showed that 17 variants had a two-fold higher risk of severe COVID-19, while 67 variants were associated with less severe COVID-19^37^. This is in line with another study from France and the US, suggesting that different viral variants may result in different infection severity and risk of hospitalization^38,39^. Since SARS-CoV-2 is an RNA virus, its dynamic evolution is expected to influence its biological characteristic^40^, including its virulence and pathogenicity^37^. Interestingly, as an RNA virus, the critical aspect of the SARS-CoV-2 life cycle is not implied by the protein sequence^41^. Indeed, one study showed the importance of synonymous substitutions on the selection of SARS-CoV-2^42^.

All our samples, except one, contained the D614G variant. Indeed, almost all viruses circulating globally consist of the D614G mutation^35^. It has been shown that the D614G mutation was not associated with the COVID-19 illness^43,44^. A large-scale analysis of the COVID-19 Genomics UK consortium demonstrated that although D614G mutation is associated with higher viral loads, it is not associated with clinical severity and fatality of COVID-19 patients^44^. Another UK study also found no association between VOC-defining mutations with the severity of COVID-19 disease^45^.

Additionally, among 123 chronically shedding immunocompromised patients, no B.1.1.7 VOC-defining mutations were detected^45^. We also observed other S protein mutations in our samples, including L5F, V213A, and S68SR. None of the mutations lies on the receptor-binding domain (RBD) of the S protein.

Interestingly, a previous report showed that one variant in non-RBD S protein, V1176F, might lead to RBD-ACE2 binding changes and was associated with a high mortality rate of COVID-19^46^. Moreover, a recent study revealed that variants within the different proteins of SARS-CoV-2 had been associated with different patients' outcomes^47^. Further in vitro experiments and population studies are essential to clarify whether multiple non-RBD S protein variants associate with the severity of COVID-19 patients. Altogether, determining the effects of single or multiple mutations within or outside the S protein on COVID-19 severity and fatality requires caution. It cannot be inferred from in vitro laboratory experiments.

There are several limitations of our study. First, we have only a limited sample size that may result in bias in our analysis. Second, the S protein continuously evolves, resulting in new mutation(s) that may significantly affect virulence and disease pathogenesis. Third, we only analyzed mutations located within the S protein. Mutations in other structural and non-structural proteins may significantly influence the COVID-19; however, they are not investigated in our study. Fourth, our study only investigated some prognostic factors affecting the COVID-19 outcomes by overall means without considering other factors, including the vaccination status. In addition, the last clinical sample in this study was collected on December 27, 2020, while the COVID-19 vaccination program was started in our country on January 13, 2021. Thus, we suggest that the mutations on the S protein of SARS-CoV-2 were more likely due to natural selection during multiple replications among hosts.

## Conclusions

Here, our study shows that hypertension and anticoagulant therapy have a substantial impact on the COVID-19 outcomes. Moreover, we suggest the possible association between SARS-CoV-2 mutations within the S protein besides the VOC with COVID-19 outcomes. Our study further suggests the importance of genomic surveillance to monitor SARS-CoV-2 variants, particularly those that might influence the outcomes of COVID-19 patients.

## Supplementary Information

Supplementary Information.

## Data Availability

All data generated or analyzed during this study are included in the submission. The sequence and metadata are shared through GISAID (www.gisaid.org).

## Abbreviations

CI: Confidence interval
OR: Odds ratio
S protein: Spike protein
SNPs: Single nucleotide polymorphisms
VOC: Variant of concern
VOI: Variant of interest
